# Serial Cryosectioning for the Spatial Transcriptomics of Plant Tissues

**DOI:** 10.21769/BioProtoc.5844

**Published:** 2026-10-20

**Authors:** Chi-Chih Wu, Kun-Ting Hsieh, Chun-Ping Yu, Yi-Hua Chen, Shu-Jen Chou, Ting-Ying Wu, Chin-Min Kimmy Ho, Shu-Hsing Wu, Mei-Yeh Jade Lu, Wen-Hsiung Li

**Affiliations:** 1 Biodiversity Research Center, Academia Sinica, Taipei, Taiwan; 2 Institute of Plant and Microbial Biology, Academia Sinica, Taipei, Taiwan; 3 Research Center for Information Technology Innovation, Academia Sinica, Taipei, Taiwan; 4 Department of Ecology and Evolution, University of Chicago, Chicago, IL, USA

**Keywords:** Spatial transcriptomics, 3D gene expression, Maize leaf development, Developmental trajectory, Transcription factor networks, Growth-regulating factors

## Abstract

Spatial transcriptomics enables genome-wide gene expression profiling while preserving tissue architecture, making it a powerful approach for studying plant developmental transitions. However, preparing small and structurally complex plant tissues for spatial transcriptomics remains technically challenging because samples must be rapidly preserved, precisely oriented, serially sectioned, and accurately positioned within the limited capture area of the Visium slides. Here, we describe an optimized workflow for cryo-embedding, serial cryosectioning, section placement, and data analysis of small plant samples for 10x Genomics Visium spatial transcriptomics. Using maize seedling shoot apices as the target, this protocol includes preparation of custom molds for optimal cutting temperature embedding, rapid fresh sample embedding, serial cryosectioning, section-position marking for Visium HD workflows, and morphological quality assessment of replicate tissue slides before transcript capture. The associated data analysis workflow includes Space Ranger processing, Seurat-based normalization and Harmony integration, anatomical domain annotation, pseudobulk and developmental trend analyses, RNA velocity, pseudotime analysis, transcription factor network analysis, single-cell reference mapping, and 3D transcriptome reconstruction. This computational workflow was developed and tested using maize Visium V1 data, but not Visium HD data. This protocol was used to generate serial spatial transcriptomes of maize shoot apices and developing leaf primordia, enabling reconstruction of gene expression transitions from the shoot apical meristem to sequential leaf developmental stages. The approach is also applicable to other small plant tissues, including *Arabidopsis* first true leaves and *Marchantia* thalli.

Key features

• Optimized cryo-embedding and cryosectioning workflow for small plant tissues for 10x Visium spatial transcriptomics.

• A streamlined computational workflow for serial-section processing, spatial-domain annotation, 3D reconstruction, and developmental trajectory analysis.

## Graphical overview


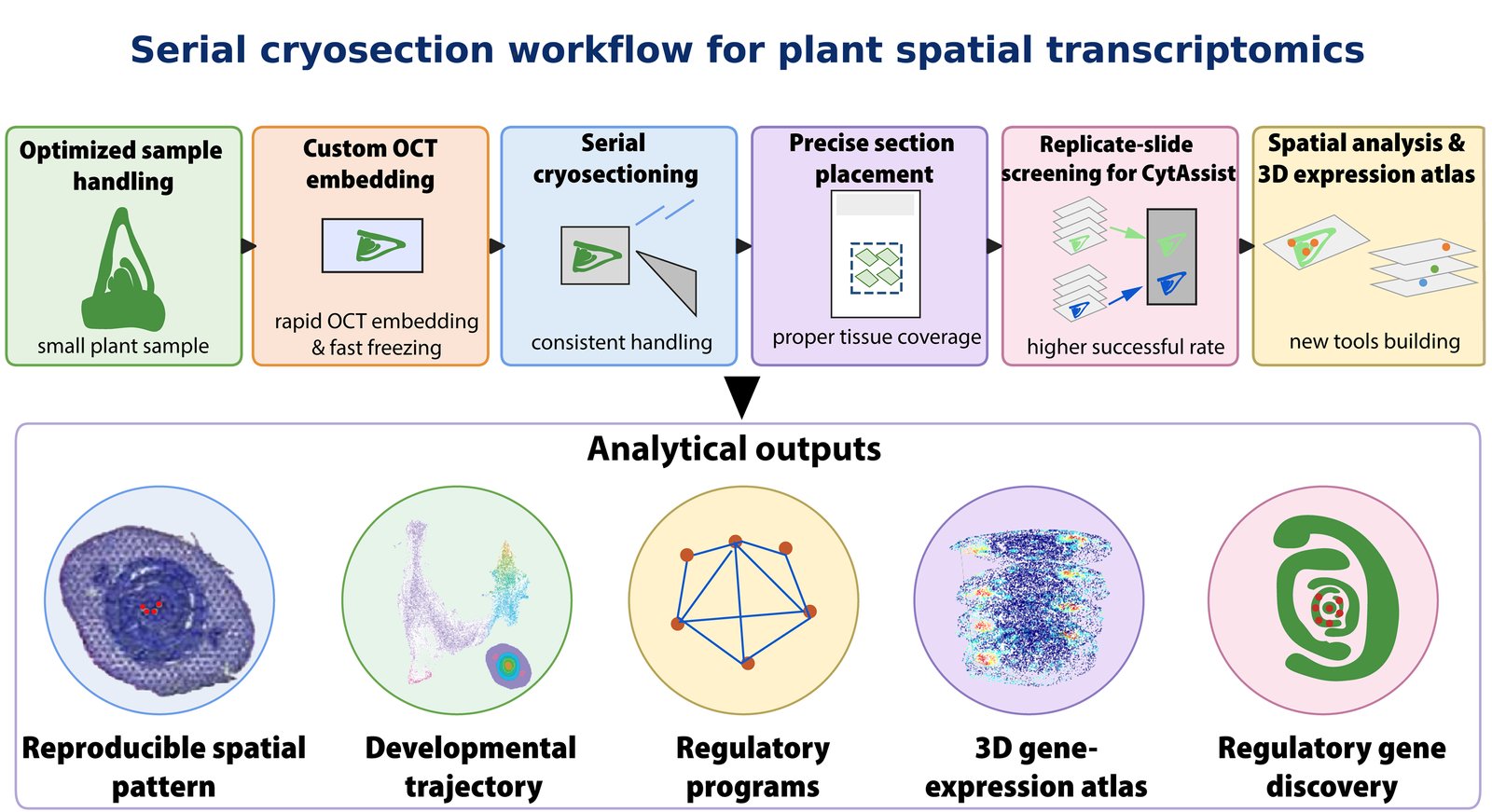


**Small plant tissues are processed using an optimized cryo-embedding and serial cryosectioning workflow for plant spatial transcriptomics.** Samples are embedded in custom optimal cutting temperature (OCT) embedding molds, rapidly frozen, cryosectioned into serial sections, and positioned within a marked Visium capture area. Replicate tissue slides are screened for tissue integrity, morphology, and placement before 10x Visium CytAssist transcript transfer. The resulting spatial transcriptomes are analyzed to generate reproducible spatial maps, reconstruct developmental trajectories, identify regulatory programs and candidate regulatory genes, and build a 3D gene expression atlas.

## Background

Spatial transcriptomics enables transcriptome-wide gene expression profiling while preserving the spatial organization within tissue sections. In plants, this approach is particularly useful for studying developmental transitions that occur across small and structurally complex tissues, such as shoot apices, leaf primordia, vascular tissues, and reproductive organs [1–4]. However, compared with animal and clinical tissues, plant samples often require additional optimization because their small size, extensive intercellular air spaces, tissue fragility, and rigid cell walls make cryosectioning difficult.

Several plant spatial transcriptomics studies have demonstrated the value of spatially resolved gene expression analysis in species such as *Arabidopsis*, poplar, barley, and maize using a species-agnostic approach [4–7]. These studies showed that spatial transcriptomics can reveal tissue-specific expression patterns and developmental gene expression gradients. However, many published plant applications [4–7] provide limited practical detail on the sample preparation steps that strongly affect data quality, such as fresh-tissue handling, optimal cutting temperature (OCT) embedding medium infiltration, freezing, tissue orientation, section attachment, and accurate placement of small sections within the capture area. These steps are particularly important for small plant samples, where excess OCT, poor orientation, air bubbles, tissue folding, or inaccurate placement can negatively impact tissue morphology, RNA quality, and transcript capture efficiency.

In our previous study, we optimized a 10x Visium-based workflow for serial spatial transcriptomics of maize seedling shoot apices [1]. The method combined rapid fresh-sample handling, OCT immersion before freezing, custom OCT embedding molds, serial cryosectioning, and computational reconstruction of 3D gene expression profiles. Using this workflow, multiple maize shoot cross-sections were placed within one 6.5 × 6.5 mm Visium capture area, enabling spatial transcriptome profiling of 14 biological replicates of shoot apical meristems with developing embryonic leaves, comprising 54 serial sections. Tissue-covered spots were annotated into seven structural domains: SAM, P1_P2, P3, P4, P5, coleoptile, and coleoptile vein.

The protocol described here expands on the experimental details of this workflow and facilitates its broader application to spatial transcriptomics studies of small plant tissues, including *Arabidopsis* first true leaves and *Marchantia* thalli. By providing a practical and integrated approach to tissue preparation, this protocol enables the generation of high-quality sections suitable for spatial transcriptomics and downstream 3D gene expression analysis.

## Materials and reagents

**Biological materials**

1. Seeds of the maize cultivar *Zea mays* cv. White Crystal used in this study were purchased from the local breeder Fong Tien Seed Co. Ltd. (Taiwan). To obtain seedlings with straight, upright stems, seeds were positioned vertically between repeatedly S-folded wet filter paper towels in Magenta^TM^ vessels (Figure 1). Seeds were grown in a growth chamber at 30 °C during the day and 26 °C at night under a 14/10 h light/dark cycle. After 72 h of imbibition, developing leaves containing the coleoptile and shoot apical meristem were carefully dissected and separated from the starchy endosperm (red dashed box in Figure 1C).

**Figure 1.**
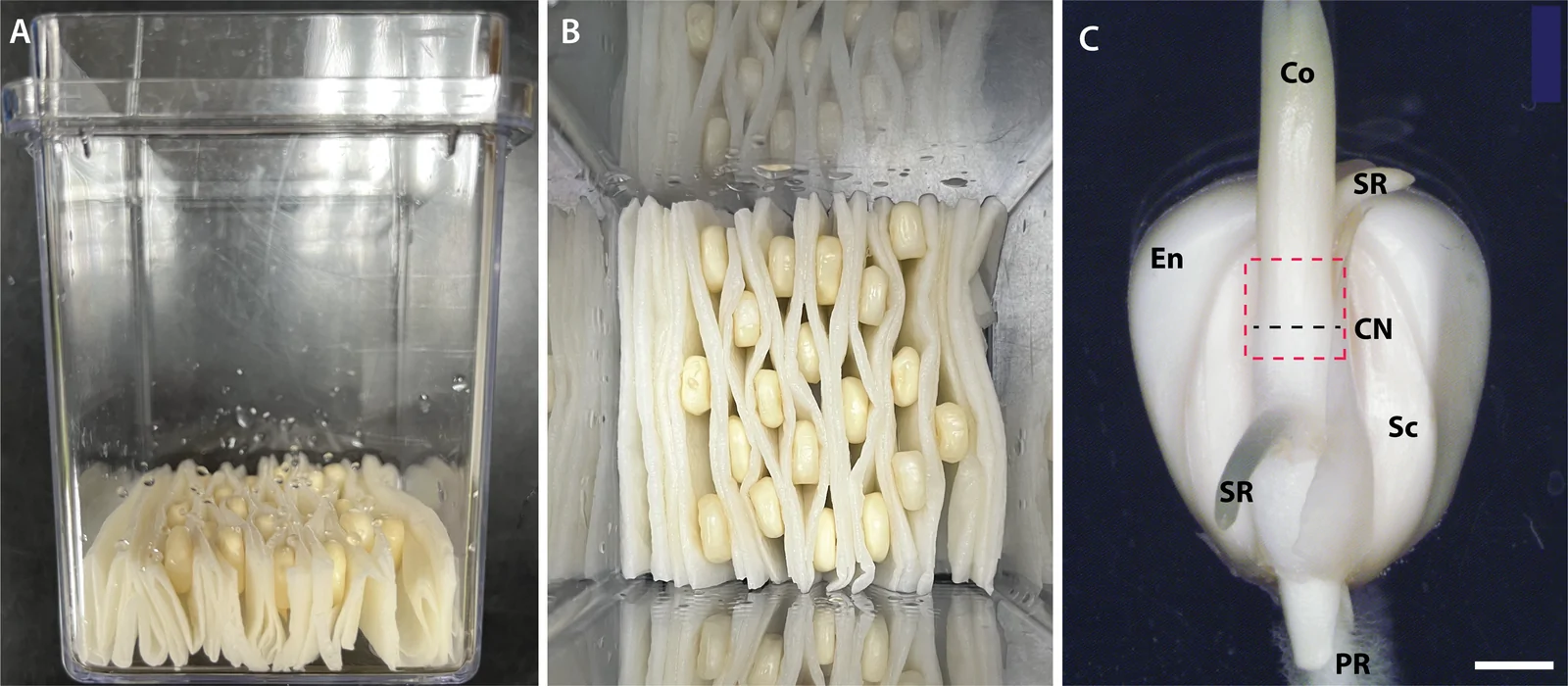
Preparation and sampling of germinating maize seedlings. (A) Maize kernels were positioned upright between folded wet paper towels in Magenta^TM^ vessels to promote straight seedling growth. (B) Top view of seed arrangement. (C) Developing seedling attached to a kernel after 72 h of imbibition, showing roots, endosperm, and coleoptile and embryonic leaf tissues enclosed within the coleoptile. The red dashed box indicates the target region for tissue collection and embedding, and the black dashed line marks the approximate section collection position at the coleoptile node. Co: coleoptile; CN: coleoptile node; En: endosperm; PR: primary root; Sc: scutellum; SR: seminal root. Scale bar, 2 mm.

**Reagents**

1. Ribonucleoside vanadyl complex 100% (New England Biolabs, catalog number: S1402S)

2. Tissue-Tek OCT 100% (Sakura, catalog number: 4583)

3. RNaseZap RNase decontamination solution (Thermo Fisher, catalog number: AM9780)

4. Isopentane (Alfa Aesar, catalog number: AF-19387-500ML)

5. Phosphate-buffered saline (PBS) (Thermo Fisher, catalog number: AM9624)

6. UltraPure DNase/RNase-free distilled water (Thermo Fisher, catalog number: 10977015)

7. Methanol, molecular biology grade (Fisher Scientific, catalog number: 34860-1L-R)

8. Isopropanol, molecular biology grade (Fisher BioReagents, catalog number: BP2618-4)

**Solutions**

1. Embedding transition medium 1 (ETM1) (20% OCT) (see Recipes)

2. Embedding transition medium 2 (ETM2) (50% OCT) (see Recipes)

3. Embedding transition medium 3 (ETM3) (100% OCT) (see Recipes)

4. Sample handling buffer (SHB) (see Recipes)

**Recipes**

**1. ETM1**

**Table:** 

Reagent	Final concentration	Quantity or volume
Milli-Q H_2_O		4 mL
OCT	20%	1 mL
Total		5 mL

**2. ETM2**

**Table:** 

Reagent	Final concentration	Quantity or volume
Milli-Q H_2_O		2.5 mL
OCT	50%	2.5 mL
Total		5 mL

**3. ETM3**

**Table:** 

Reagent	Final concentration	Quantity or volume
Milli-Q H_2_O		0 mL
OCT	100%	5.0 mL
Total		5.0 mL

**4. SHB**

**Table:** 

Reagent	Final concentration	Quantity or volume
1× PBS	90%	4.5 mL
Ribonucleoside vanadyl complex	10%	0.5 mL
Total		5.0 mL

**Laboratory supplies**

1. Magenta^TM^ vessel GA-7 (Sigma-Aldrich, catalog number: V8505)

2. Laboratory folded paper towels (Scott, catalog number: 60055)

3. Fisherbrand Superfrost Plus microscope slides (Fisher Scientific, catalog number: FIS12-550-15)

4. Dumont Tweezers Positive Action 3C (Electron Microscopy Sciences, catalog number: 50-949-284)

5. Dissecting scissors (Fisher Scientific, catalog number: 08-940)

6. Hypodermic needles (19G × 1 1/2") (Terumo, catalog number: B130022)

7. Aluminum foil (Diamond Crystal, catalog number: 75 SQFT)

8. Falcon conical 50 mL high clarity PP centrifuge tube (Corning, catalog number: 352098)

9. 60 × 15 mm plastic Petri dishes (Corning, catalog number: BP53-03)

10. 35 × 15 mm plastic Petri dishes (Fisher Scientific, catalog number: 50-147-469)

11. Lint-free laboratory wipes (Texwipe Vectra Alpha 10, catalog number: TX1010)

12. Reagent reservoirs (Corning Costar, sterile disposable reagent reservoirs, catalog number: 07-200-130)

13. Liquid nitrogen or dry ice (local provider)

14. Slide mailer (Fisher Scientific, catalog number: HS15986)

15. Razor blades (Merkur)

16. Single-edge safety razor (Educlub, catalog number: MV-ED0068A)

17. Metal containers for frozen sample transfer (accessories of Leica EMPACT2)

## Equipment

1. Growth chamber (Firstek, model: S2703-GC)

2. Freezer (-20 °C) (Nihon Freezer, model: SCF-FG-4002)

3. Freezer (-80 °C) (Nihon Freezer, model: CLN-52UWT)

4. Refrigerator (4 °C) (Nihon Freezer, model: SCF-FG-4002)

5. Dissecting microscope (Nikon, model: SMZ)

6. Rotary pump (Edwards, model: E2S45)

7. Histology vacuum chamber (Tarsons, model: T403030)

8. Brightfield microscope (Evident, model: CX33)

9. Cryostat (Leica, model: CM 1950)

10. Thermal cyclers (Thermo Fisher Scientific, model: VeritiPro Thermal Cycler A48141)

## Software and datasets

1. Space Ranger (v2.1.0 for Visium or v4.1.0 for Visium HD 3’, 10x Genomics, https://www.10xgenomics.com/support/software/space-ranger/latest/release-notes/release-notes-for-SR)

2. Loupe Browser (v9.1, 10x Genomics, https://www.10xgenomics.com/support/software/loupe-browser/latest)

3. R environment (v4.6.1, https://www.r-project.org/about.html)

4. Python (v3.11.16, https://www.python.org/)

5. Seurat (v5.5.1, Satija Lab and Collaborators, https://satijalab.org/seurat/)

6. STUtility (v1.1.1, GRG, KTH, https://ludvigla.github.io/STUtility_web_site/)

7. SeuratWrappers (v0.4.0, a collection of Harmony v2, Monocle 3, scVelo v0.2.5, https://github.com/satijalab/seurat-wrappers)

8. SCTransform (v0.4.3, https://github.com/satijalab/sctransform)

9. Velocyto (v0.17.17, https://velocyto.org/velocyto.py/tutorial/index.html#running-the-cli)

10. scDblFinder (v1.26.7, https://github.com/plger/scDblFinder)

11. SCINA (v1.2.0, https://github.com/jcao89757/SCINA)

12. SPOTlight (v1.16.0, https://github.com/MarcElosua/SPOTlight)

13. TO-GCN (https://github.com/petitmingchang/TO-GCN)

14. glmGamPoi (v1.24.0, https://github.com/const-ae/glmGamPoi)

15. Harmony (v2.0.5, https://github.com/immunogenomics/harmony)

16. scVelo (v0.3.4, https://scvelo.readthedocs.io/en/stable/)

17. SeuratObject (v5.4.0, https://github.com/satijalab/seurat-object)

18. Monocle 3 (v1.4.27, https://cole-trapnell-lab.github.io/monocle3/)

19. Napari (v0.7.1, https://napari.org/stable/)

20. Maize shoot data-processing pipeline (https://github.com/bomacchih/maize_shoot_data_process_v2)

21. Maize reference genome (Zm-B73-REFERENCE-NAM-5.0.fa)

22. Maize gene annotation (Zm-B73-REFERENCE-NAM-5.0.51.gtf)

23. Maize shoot scRNA-seq datasets (SRA: SRR11943512 and SRR11943513)

24. Maize shoot Visium datasets (PRJNA805024 and PRJNA804974)

25. Processed datasets for Napari interactive viewer on Zenodo (https://zenodo.org/records/16933147)

26. Processed datasets for bioinformatic analyses on Zenodo (https://zenodo.org/records/22058284)

27. agriGo v2.0 (https://systemsbiology.cau.edu.cn/agriGOv2/)

## Procedure

**A. Prepare custom OCT embedding molds**

Optimize the mold size according to the sample size but avoid making the mold excessively large. Oversized molds require more OCT, increase the time needed for OCT solidification, and make block handling and trimming less efficient. In addition, excess OCT may reduce the efficiency of rapid freezing, increasing the risk of ice crystal formation inside the tissue. Most commercially available sample-positioning tools are designed for animal or clinical specimens, which are generally much larger than many plant samples. Therefore, these tools are often not well-suited for small plant tissues, such as maize shoot apices, *Arabidopsis* first true leaves, or *Marchantia* thalli.

For small plant samples, an embedding mold with internal dimensions approximately three times those of the specimen provides a practical starting point, although this is not a strict requirement. The mold should allow sufficient space for specimen handling, orientation, and complete coverage with OCT while minimizing the total OCT volume. In our experiments, the approximate internal mold dimensions (length × width × height) were 6 × 5 × 5 mm for maize seedling shoots, 8 × 6 × 6 mm for *Arabidopsis* first true leaves, and 8 × 7 × 6 mm for *Marchantia* thalli. These dimensions can be adjusted according to the size and shape of the specimen (Figure 2).

1. Prepare sheets of aluminum foil: Start with a flat rectangular sheet of aluminum foil (Figure 2A).

2. Fold in half: Fold the sheet along the center line (Figure 2A) to make a smaller double-layer rectangle (Figure 2B).

3. Add fold lines: Mark two vertical lines, two horizontal lines, and four diagonal corner lines (Figure 2C).

4. Form the boat structure (Figure 2C, D).

a. Fold the side walls: Fold the left and right panels inward along the vertical lines.

b. Fold the front and back walls: Fold the top and bottom panels upward along the horizontal lines.

c. Pinch the corners: Fold the four corners along the diagonal lines to form triangular corner supports.

d. Open the center: Gently open the middle area to create a shallow container.

e. Adjust the shape: Press the walls and corners until the final structure forms a small, rectangular, boat-like container.

**Figure 2.**
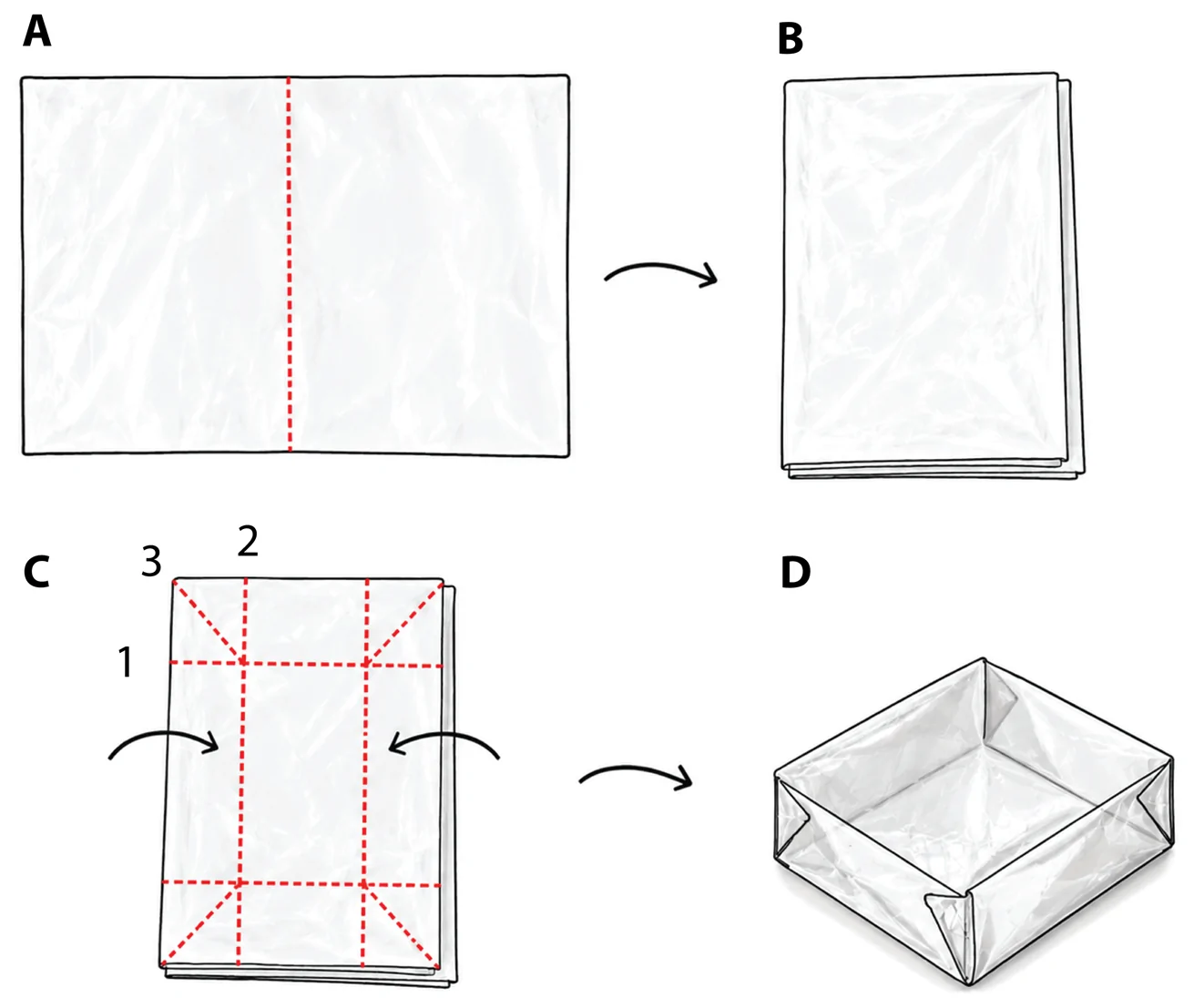
Preparation of a custom OCT embedding mold. Following steps A–D, a thin sheet of aluminum foil is folded stepwise to generate a boat-like container for sample embedding. Numbers 1–3 indicate the order of folding: 1, side walls; 2, front and back walls; 3, corner folds.

**B. Build a custom section-position guide tool**

For 10x Visium HD workflows, tissue sections are first placed on compatible blank tissue slides and are later transferred to the Visium HD slide using the 10x Visium CytAssist instrument. The 10x Genomics handbook recommends drawing an outline of the allowable area on the back of the blank slide before section placement to ensure compatibility with the CytAssist instrument and refers users to the Visium CytAssist Tissue Slide Alignment Instruction Quick Reference Cards (CG000548) for determining this allowable area [8].

Although 10x Genomics provides the optional Visium CytAssist Alignment Aid for marking the back of tissue slides [8], this tool still requires manual slide positioning and marking, and it does not hold each slide in a fixed, reproducible position during preparation. In practice, this can make it difficult to mark the same target region consistently across multiple slides, especially when preparing replicate plant tissue sections for screening. Therefore, we designed a custom section-position guide tool to hold standard microscope slides in a fixed position and to mark a consistent target area for section placement. This guide improves reproducibility when positioning small plant tissue sections within the CytAssist-compatible allowable area.

*Note: If using 10x Visium V1, skip this section, because cryosections are placed directly onto Visium Gene Expression slides.*

1. Prepare the guide tool (Figure 3A)

a. Build two cardboard parts: a bottom part and a top part.

b. Both parts should be aligned to the same center line (blue dashed line).

c. The bottom part contains a small 8 × 8 mm square window for marking the target section area.

*Note: For the 11 × 11 mm version, prepare an additional cardboard template with a 12 × 12 mm square window.*

d. The top part contains a long central opening to hold and align the microscope slide.

**Figure 3.**
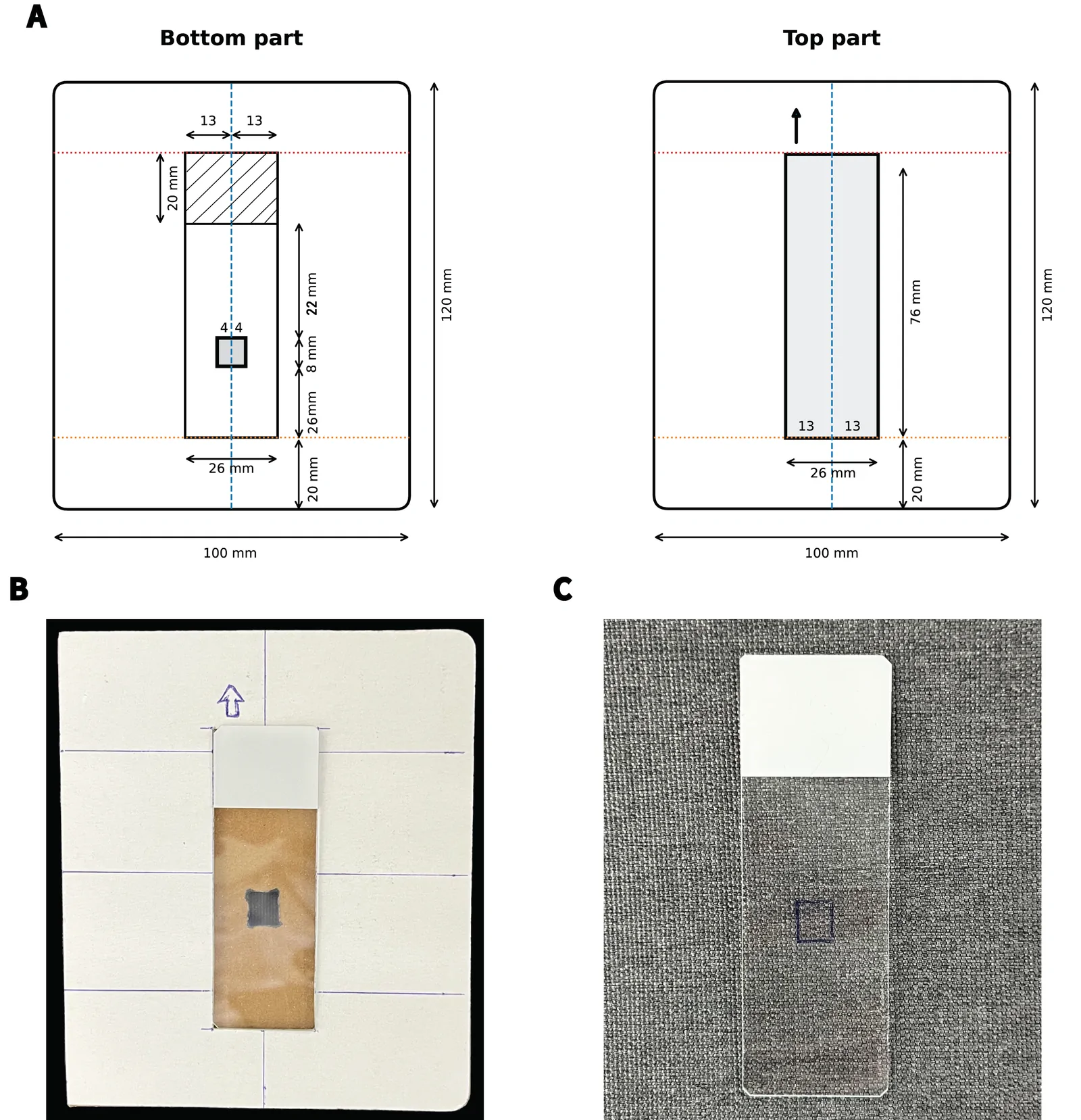
Section-position guide tool for 10x Visium V2 or HD slide preparation. (A) Design layout of the section-position guide tool, consisting of a bottom part and a top part. The guide is centered on a 100 × 120 mm cardboard template, with a central 26-mm-wide slot and an 8 × 8 mm marking window aligned to the center line. Gray areas are cut out. (B) Assembly of the guide tool. The top and bottom parts are aligned, and the microscope slide is placed face-up between them. The 8 × 8 mm square is marked from the back side of the slide through the guide window. (C) Microscope slide after marking, showing the 8 × 8 mm section-position reference square (in blue) used to guide tissue placement. Sections are placed within the square.

2. Assemble the guide and mark the section position (Figure 3B)

a. Place the bottom part on a flat surface.

b. Put the microscope slide face-up on the bottom part.

c. Align the slide with the center line and guide lines.

d. Place the top part over the slide and align it with the bottom part.

e. Invert the assembled guide so the back side of the slide can be accessed.

f. Use the 8 × 8 mm window on the bottom guide as the reference.

g. Draw the 8 × 8 mm square on the back side of the slide using a marker.

3. Use the marked slides: The marked 8 × 8 mm square indicates the target tissue placement area (Figure 3C).

**C. Preparation of cryo-embedded fresh samples**

*Note: To minimize RNA degradation, keep all equipment, reagents, and tools on ice throughout sample preparation. Complete the entire sample handling procedure within 5 min.*

1. Prepare equipment and reagents (Figure 4)

a. Prepare a tube of SHB (see Recipes) and keep it on ice.

b. Prepare three 35 mm Petri dishes containing ETM1–ETM3 solutions (see Recipes). Place the dishes on ice in the order of use (Figure 4B).

c. Prepare isopentane pre-chilled with liquid nitrogen or dry ice (Figure 4C).

d. Place OCT embedding boats in a 60 × 15 mm Petri dish on ice. Mark the desired sample orientation on the side of each boat and fill each boat with pre-cooled OCT.

**Figure 4.**
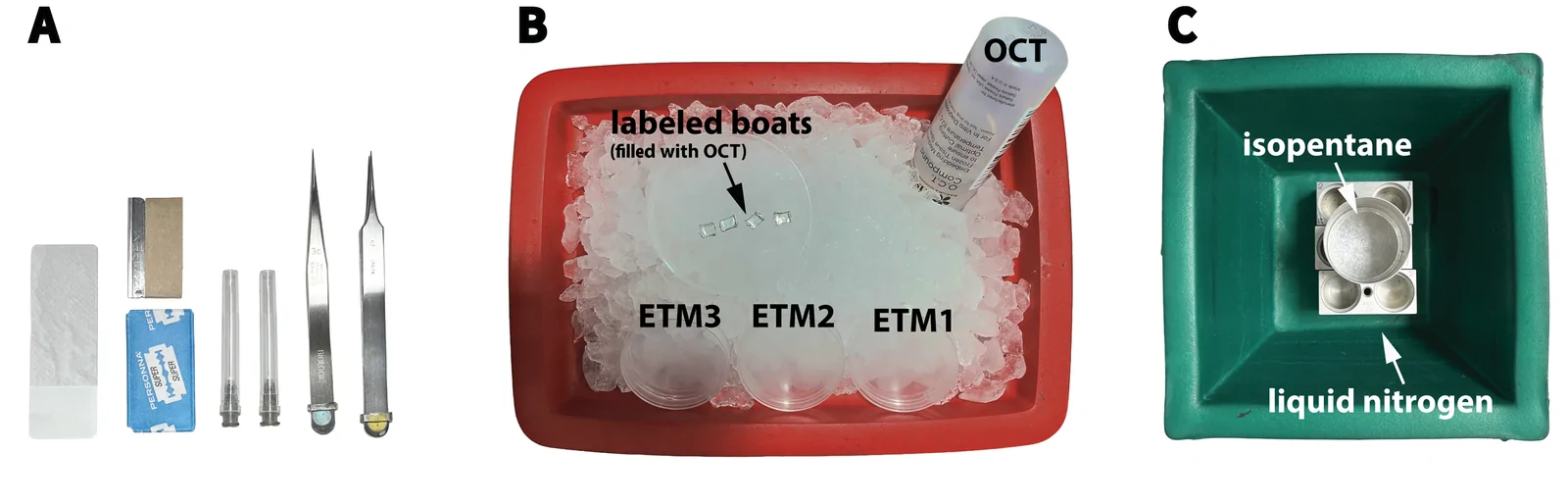
Preparation of equipment and reagents for cryo-embedding fresh plant samples. (A) Tools used for fresh sample handling and orientation, including razor blades, needles, and fine forceps. All tools should be prepared in advance and kept cold when possible to minimize RNA degradation. (B) Ice-cold setup for sample embedding, including ETM1–3 in Petri dishes, OCT embedding boats, and pre-cooled OCT. ETM1–3 are placed on ice in the order of use to allow rapid transfer of dissected samples through 20%, 50%, and 100% OCT. (C) Pre-chilled isopentane setup for rapid freezing of OCT-embedded samples. Metal containers for isopentane or frozen sample transfer are accessories of a Leica EMPACT2. After sample orientation in OCT-filled embedding boats, the boats are immediately immersed in pre-chilled isopentane until the OCT is completely solidified. Embedded blocks are then stored at -80 °C until cryosectioning.

2. Embed fresh samples in OCT

a. Dissect the maize seedlings in ice-cold SHB.

b. Transfer the dissected samples into a bottle containing fresh SHB.

c. Place the bottle in a histology vacuum chamber connected to a vacuum pump.

d. Apply vacuum until trapped air is removed from the samples, usually within 1 min.

**Critical:** Vacuum infiltration is particularly important for plant tissues because air spaces can prevent complete OCT infiltration and reduce section quality.

e. Sequentially transfer the samples through ETM1 (20%), ETM2 (50%), and ETM3 (100%).

**Critical:** Minimize the incubation time in each transition medium, preferably to less than 30 s, to preserve RNA integrity while allowing sufficient infiltration.

f. Transfer the samples into OCT-filled embedding boats and adjust the sample orientation under a stereomicroscope.

**Critical:** Correct sample orientation at this step determines the final sectioning plane and cannot be corrected after freezing.

g. Immediately immerse the embedding boats in pre-chilled isopentane until the OCT is completely solidified.

h. Transfer the frozen OCT blocks to a -80 °C freezer and store them until cryosectioning.

**Pause point:** Embedded samples can be stored long-term at -80 °C before cryosectioning. Avoid repeated freeze-thaw cycles.

**D. Cryosectioning**

*Note: For 10x Visium V1, cryosections are placed directly onto the capture areas of Visium Gene Expression slides. For Visium HD probe-based or poly(A)-capture workflows, cryosections are placed onto the marked slides prepared in Section B.*

1. Set the cryostat chamber temperature to -18 °C.

2. Once the chamber reaches -18 °C, transfer the embedded samples from the freezer to the cryostat chamber and allow them to equilibrate for at least 30 min.

3. Remove the aluminum embedding boat and mount the specimen block onto a cryostat specimen chuck according to the desired cutting orientation (Figure 5A).

4. Apply a small amount of OCT around the base of the specimen block to secure it to the chuck.

5. Roughly trim the OCT block until the sample is exposed.

6. Mount the specimen chuck onto the orienting head of the cryostat cutting arm and adjust the sample orientation.

7. Fine-trim the block, leaving approximately 2 mm of OCT around the target sample (Figure 5B).

8. Adjust the anti-roll glass plate and set the section thickness. For plant tissues, 12–14 μm is recommended to obtain intact sections.

9. Place Visium Gene Expression slides or marked Superfrost Plus slides in the cryostat chamber for temperature equilibration for at least 20 min until -18 °C is reached before section transfer.

10. Cut one cryosection. Ideally, the section remains attached to the blade edge after cutting.

*Note: See Video 1 for the following steps.*

**Video 1.**
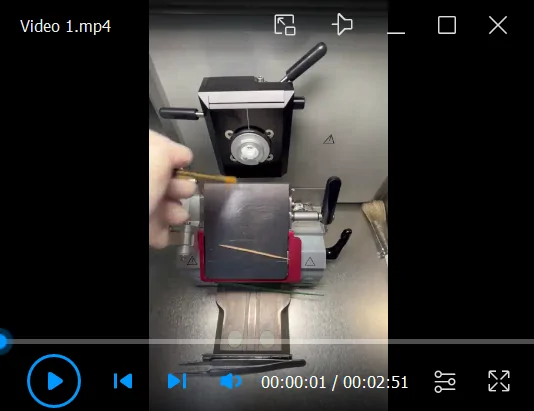
Multi-section cryosectioning within a single Visium capture area

11. Use a fine paintbrush to gently extend and flatten the cryosection on the blade edge (Figure 5C).

12. To arrange multiple sections within the target capture area, use a fine, sharpened bamboo toothpick. Gently press the side of the toothpick against either the left or right edge of the section, depending on personal preference.

13. Use the paintbrush to gently brush the blade edge and release the section from the blade (Figure 5D).

14. Firmly hold the slide and briefly warm the target capture area from the back side with a fingertip. Quickly transfer the section with the toothpick into the capture area (Figure 5E). Once the section melts and attaches to the slide, immediately place the slide back onto the metal surface inside the cryostat chamber to cool it (Figure 5F).

**Critical:** The section will attach quickly to the slide upon contact. Avoid prolonged warming, which may reduce RNA quality or cause section deformation.

15. Cut the next serial section and place additional sections within the capture area until the desired number of sections has been collected.

16. Store prepared tissue slides in a 50 mL Falcon conical tube at -80 °C until processing with the 10x Visium workflow.

**Pause point:** Slides can be stored at -80 °C for up to four weeks before downstream 10x Visium processing. Keep slides frozen and avoid freeze/thaw cycles.

**Figure 5.**
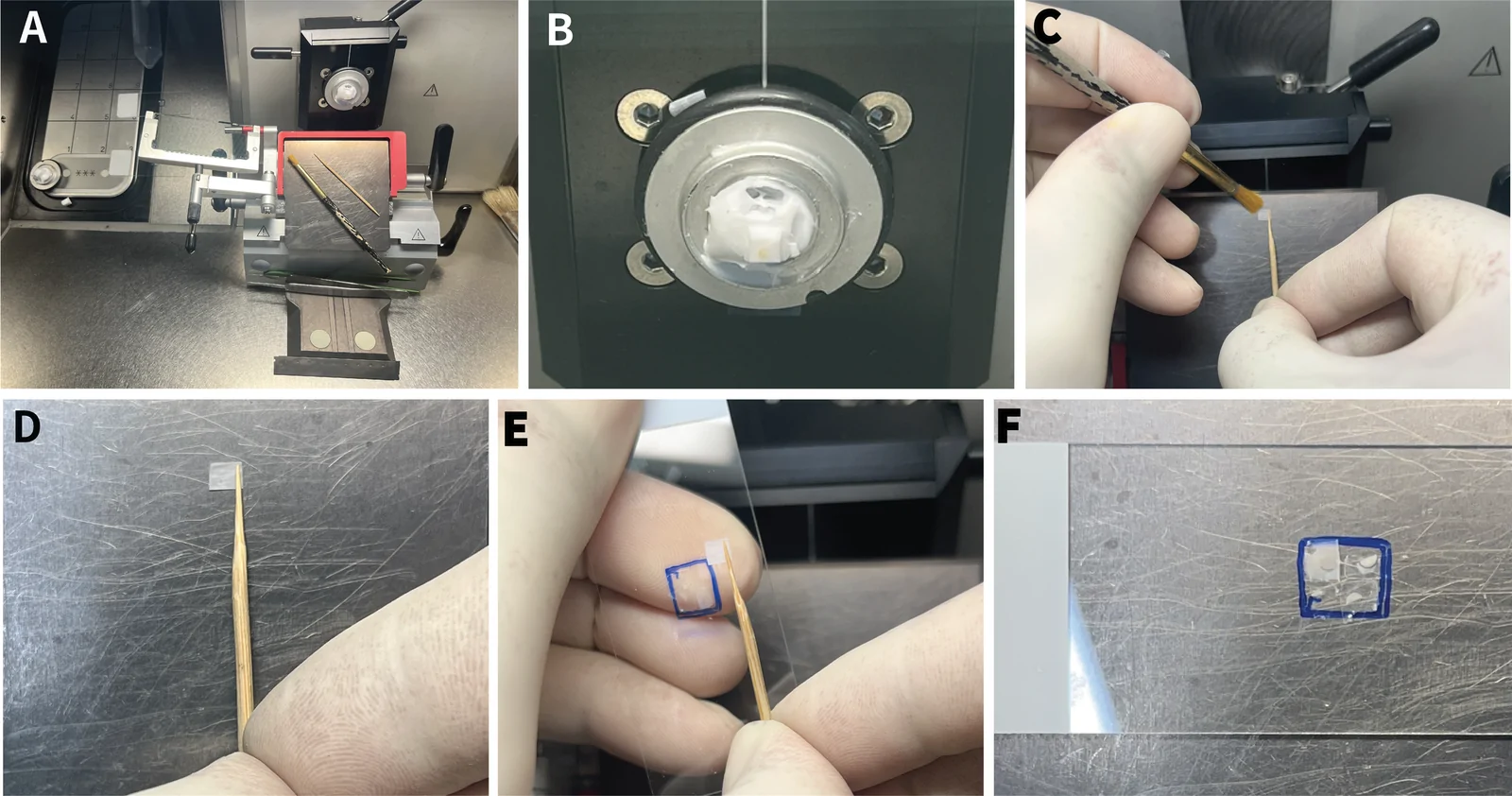
Cryosectioning and placement of serial plant tissue sections. (A) Cryostat setup for sectioning OCT-embedded plant samples. The specimen block is mounted on the cryostat chuck, and marked slides are equilibrated inside the chamber before section transfer. (B) Fine trimming of the OCT block after the sample is exposed. Approximately 2 mm of OCT is left around the target tissue to support sectioning. (C) A fine paintbrush is used to gently extend and flatten the cryosection on the blade edge. (D) A sharpened bamboo toothpick is used to contact the side of the section and assist section transfer. (E) The section is transferred to the marked capture area of the slide after briefly warming the back side of the slide with a fingertip. (F) Multiple serial cryosections are arranged within the marked capture area. After section attachment, the slide is immediately cooled in the cryostat and stored at -80 °C until downstream 10x Visium processing.

**E. Selection of the optimal tissue slide for transcript transfer and capture**

*Note: This section is not applicable to the 10x Visium V1 Gene Expression slide.*

For 10x Visium HD kits, the provided reagents and consumables limit the number of tissue slides that can be tested before the final experiment. The official workflow allows only limited additional slide screening. Plant tissue sections often vary in morphology, attachment, and placement quality. We recommend preparing additional replicate tissue slides for screening prior to the CytAssist transcript transfer workflow when sufficient tissue is available.

Based on the 10x Genomics technical recommendations for plant samples [9], the fixation step in Section 2.5 of the *10x Visium HD 3′ Fresh Frozen Tissue Preparation Handbook* can be modified to include methanol and isopropanol fixation followed by an extended air-drying period. During this air-drying step, the replicate slides can be inspected to evaluate tissue morphology, tissue attachment, and section placement. This screening step helps identify the most representative slide and ensures that only high-quality sections are used for downstream spatial transcriptomics (Figure 6).

**Figure 6.**
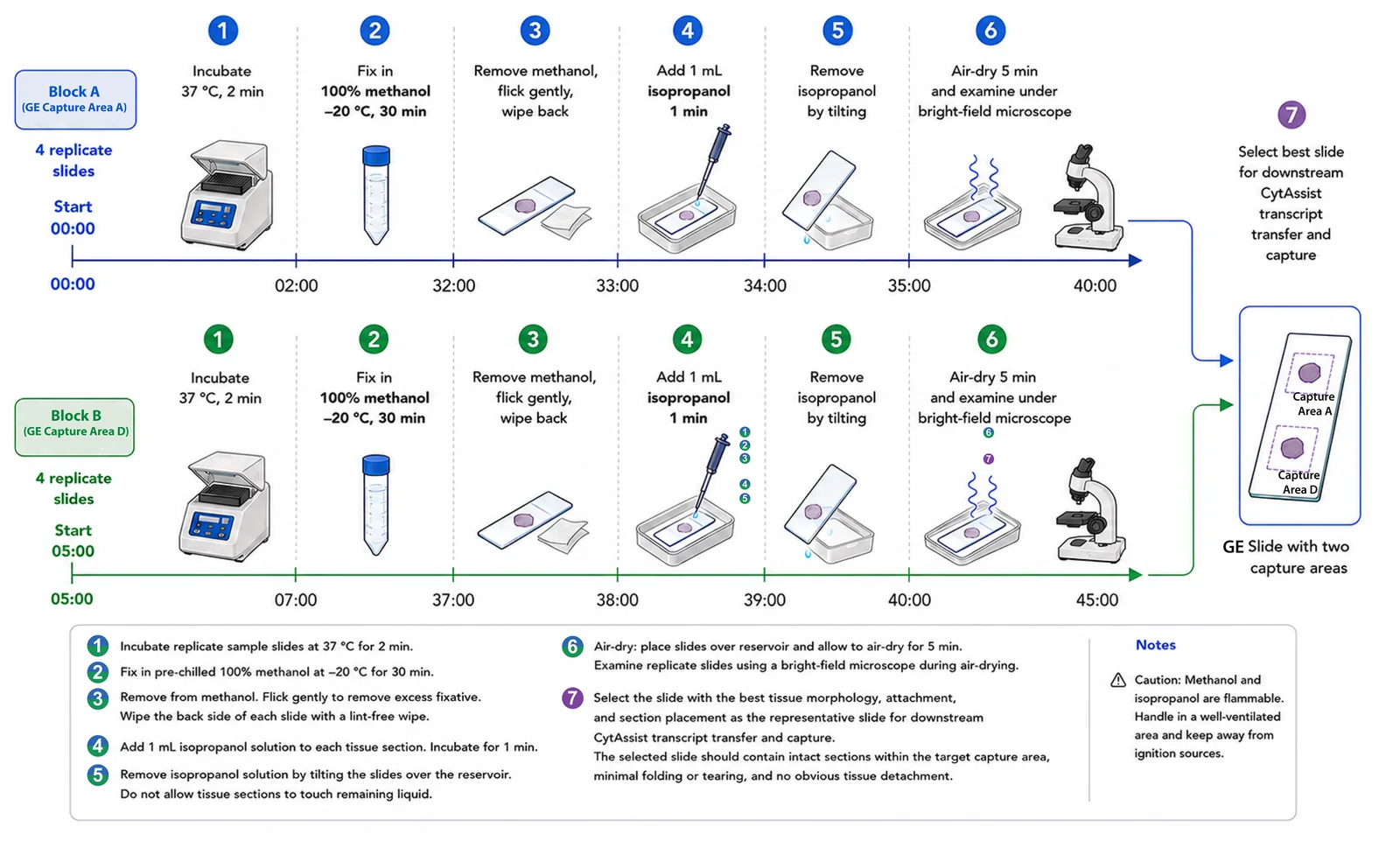
Timeline workflow for fixation and selection of replicate tissue slides. Two sets of four replicate tissue slides are processed in parallel with a 5-min staggered start time. Slides from Block A begin at 00:00, and slides from Block B begin at 05:00. Slides are first incubated at 37 °C for 2 min, fixed in pre-chilled 100% methanol at -20 °C for 30 min, briefly treated with 100% isopropanol for 1 min, and then air-dried for 5 min. During air-drying, replicate slides are examined by brightfield microscopy. The slides with the best tissue morphology and tissue attachment are selected for downstream Visium CytAssist transcript transfer and capture.

1. Fix tissue slides and select the best sample slide for CytAssist transfer.

a. Prepare 100% methanol up to 24 h in advance and store it at -20 °C.

**Caution:** Methanol and isopropanol are flammable. Handle them in a well-ventilated area and keep them away from ignition sources.

b. After incubating the replicate tissue slides in a thermal cycler at 37 °C for 2 min, immediately remove them from the thermal cycler.

c. Gently immerse the slides in a slide mailer containing pre-chilled 100% methanol and incubate them at -20 °C for 30 min.

d. Remove the slides from the methanol fixation solution. Flick the slides gently to remove excess fixative and wipe the back side of each slide with a lint-free laboratory wipe.

**Critical:** Do not touch the tissue sections on the front side of the slide.

e. Place the slides over a reagent reservoir with the tissue sections facing up.

f. Add 1 mL of isopropanol solution to each tissue section and incubate for 1 min.

g. Remove the isopropanol solution by tilting the slides over the reagent reservoir.

**Critical:** Do not allow the tissue sections to touch any remaining liquid in the reservoir.

h. Place the slides back over the reagent reservoir and allow them to air-dry for 5 min.

i. During air-drying, examine the replicate slides using a brightfield microscope.

j. Select the slide with the best tissue morphology, tissue attachment, and section placement as the representative slide for downstream CytAssist transcript transfer and capture.

**Critical:** The selected slide should contain intact sections within the target capture area, minimal folding or tearing, and no obvious tissue detachment.

*Note: Each Visium HD spatial slide contains two independent 6.5 × 6.5 mm capture zones (designated A1 and D1). Ensure that tissue positioning on the standard slides lines up with these active regions during CytAssist assembly.*

**F. Data analysis**

The data analysis workflow is summarized in Figure 7. In the original study using Visium V1 GE slides [1], serial spatial transcriptomes were generated from 14 biological replicates (capture areas) on four 10x Gene Expression slides comprising 54 maize shoot cross-sections, with three or four sections placed within each 6.5 × 6.5 mm capture area on 10× Visium V1 Gene Expression slides (Figure 8). Tissue-covered spots were annotated into seven structural domains according to the histological pattern: SAM, P1_P2, P3, P4, P5, coleoptile, and coleoptile vein (co_v). Spots overlapping two or more structural domains were excluded from domain-level analyses.

The overall analysis workflow includes raw data processing, quality control, spatial object construction, normalization and integration, anatomical domain annotation, marker-gene validation, pseudobulk analysis, gene expression trend analysis, RNA velocity, pseudotime analysis, transcription factor regulatory-network analysis, single-cell reference integration, cell-type deconvolution, and 3D visualization (Figure 7). The following section describes the basic workflow for these analyses. The detailed code is available in the GitHub repository (github.com/bomacchih/maize_shoot_data_process_v2).

*Note: Example scripts in this repository are provided for STUtility/Seurat import, section cropping, image masking, Harmony integration, marker analysis, pseudobulk comparison, Monocle 3 pseudotime analysis, metadata transfer for scVelo, SCINA annotation, SPOTlight deconvolution, and 3D visualization. Basic R knowledge is required, as some parameters must be adjusted for individual datasets. The provided code was tested using datasets generated from Visium V1. Datasets generated from Visium HD have not yet been tested; therefore, caution should be taken when applying these scripts to Visium HD data. (At the time of manuscript preparation, newer Space Ranger versions had added expanded Visium HD support. Users should follow the current 10x Genomics documentation.)*

**Figure 7.**
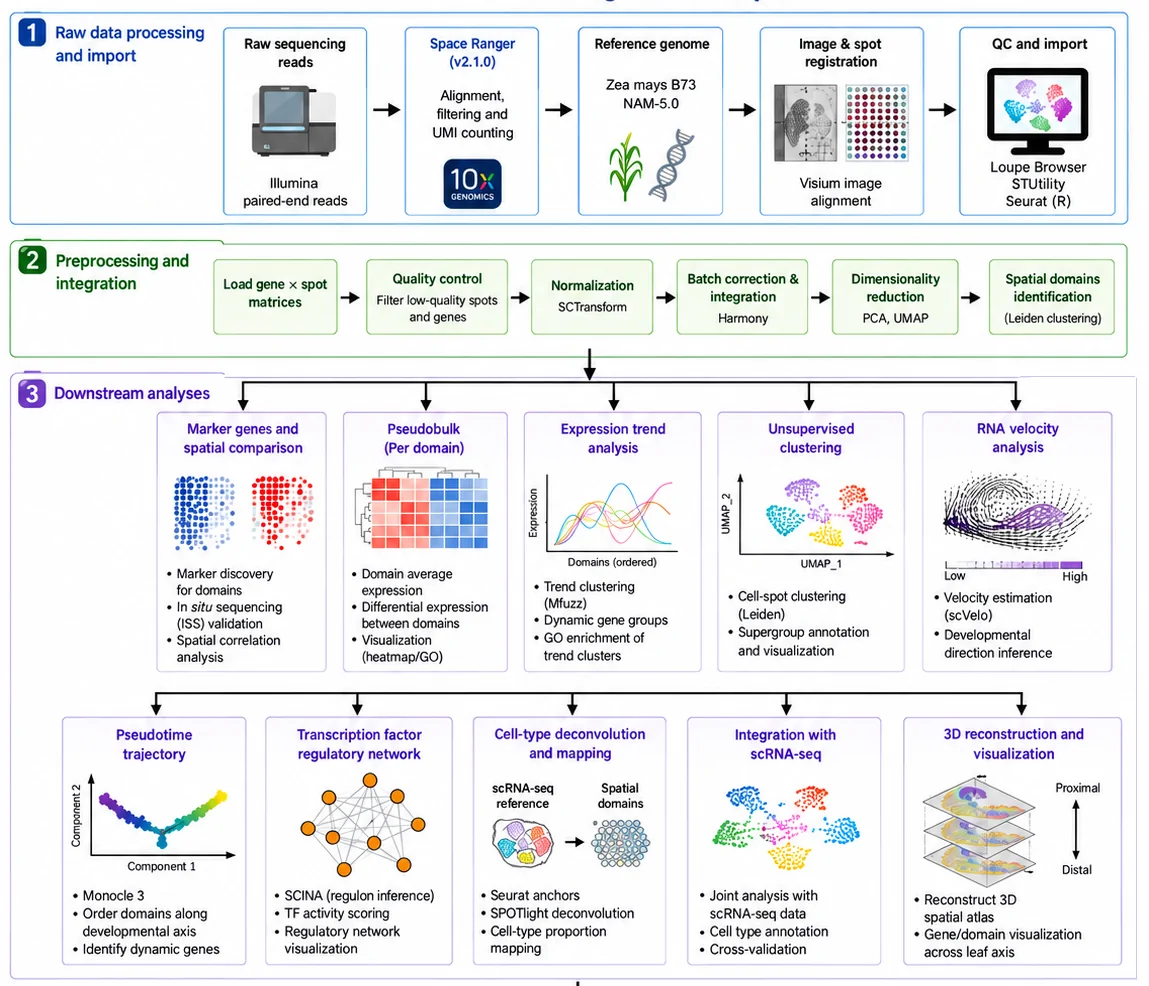
Bioinformatics workflow for serial spatial transcriptomes of maize embryonic leaf development. Raw BCL files of Illumina paired-end reads are processed using Space Ranger to generate gene-by-spot matrices, spatial barcodes, UMI counts, and image registration files. Spatial transcriptome data are imported into R, quality controlled, split by section, normalized, integrated, and annotated according to seven anatomical domains. Downstream data analyses include marker gene identification, pseudobulk comparison, expression trend clustering, GO enrichment, unsupervised clustering, RNA velocity, Monocle 3 pseudotime analysis, TO-GCN transcription factor network analysis, scRNA-seq integration, SCINA cell-type annotation, Seurat anchor label transfer, SPOTlight deconvolution, and 3D visualization using STUtility and Napari. These analyses support developmental trajectory reconstruction, identification of regulatory programs, cell/domain specialization analysis, and prioritization of candidate genes for validation.

**Figure 8.**
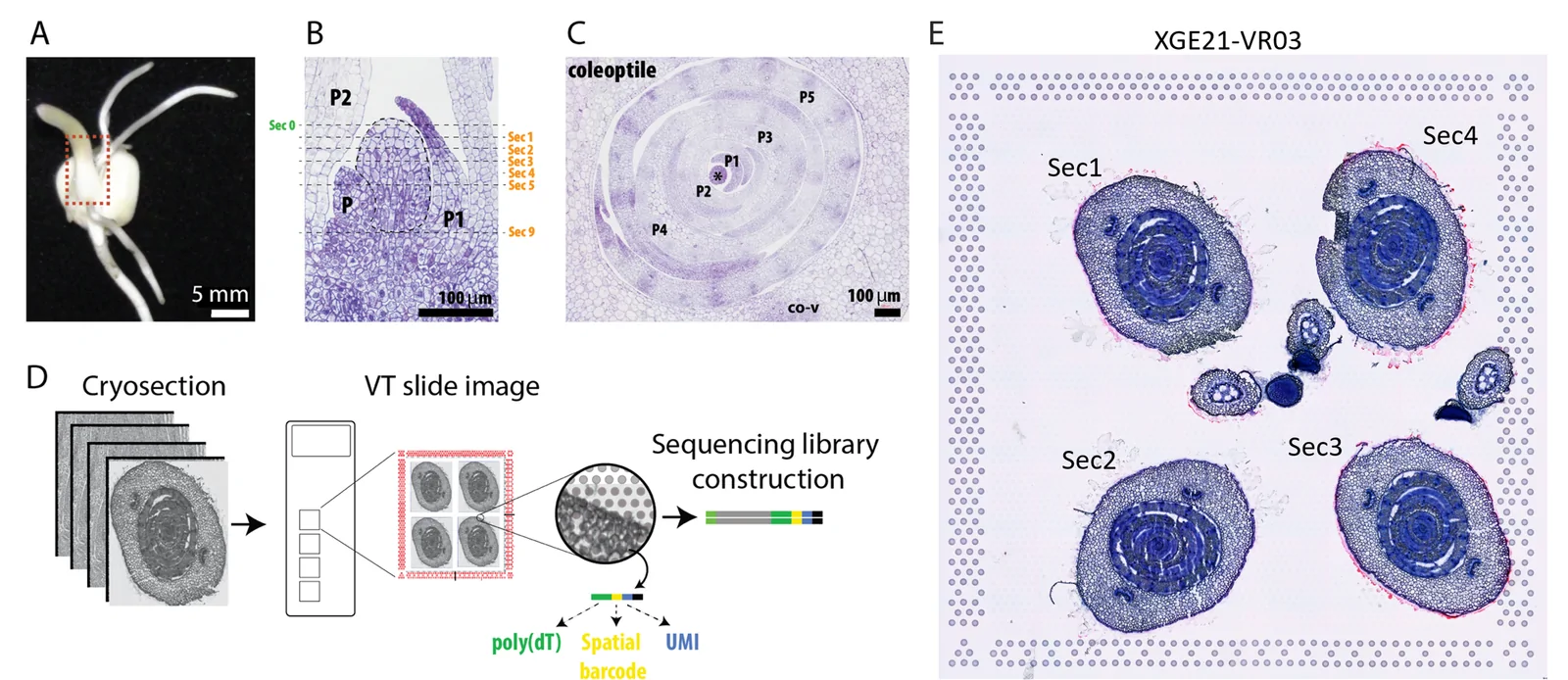
Serial sectioning and spatial transcriptomic profiling of maize shoots. (A) Maize shoot showing the sampled region (red dashed box). (B) Longitudinal section showing the positions of serial cross-sections (Sec) through the shoot apex. (C) Representative cross-section showing the coleoptile, coleoptile vein (co_v), and developing leaf primordia at successive plastochron (P) stages, P1–P5. (D) Workflow for cryosectioning, placement on a Visium slide, and spatial gene expression library construction. (E) Representative capture area containing four serial sections (Sec1–4) from sample XGE21-VR03. Scale bars: 5 mm (A) and 100 μm (B, C). Modified from Figure 1 of [1].

**F1. Process raw sequencing data using Space Ranger**

1. Raw Illumina base call (BCL) files were demultiplexed and converted into standard paired-end FASTQ files using the spaceranger mkfastq pipeline (10x Genomics).

2. Prepare a custom maize reference genome using “spaceranger mkref”. In the original study, reads were mapped to the maize Zm-B73-REFERENCE_NAM-MT-5.0.dna.toplevel reference, using the Zm-B73-Reference-NAM-5.0 genome annotation (https://www.maizegdb.org/genome/assembly/Zm-B73-REFERENCE-NAM-5.0).

3. Process each Visium capture area using “spaceranger count” with the corresponding brightfield tissue histology (e.g., toluidine blue or HE staining). Use the default settings for image alignment, tissue detection, fiducial detection, barcode counting, and UMI counting.

4. Inspect the Space Ranger outputs, including the .cloupe, web summary, tissue image alignment, filtered feature-barcode matrix, spatial coordinates, and tissue-position files (Figure 9).

5. Examine each capture area in Loupe Browser or import the output into R for downstream analysis using STUtility and Seurat.

*Note: .cloupe files and web summaries are available on Zenodo (* *https://zenodo.org/records/22058284* ).

**Figure 9.**
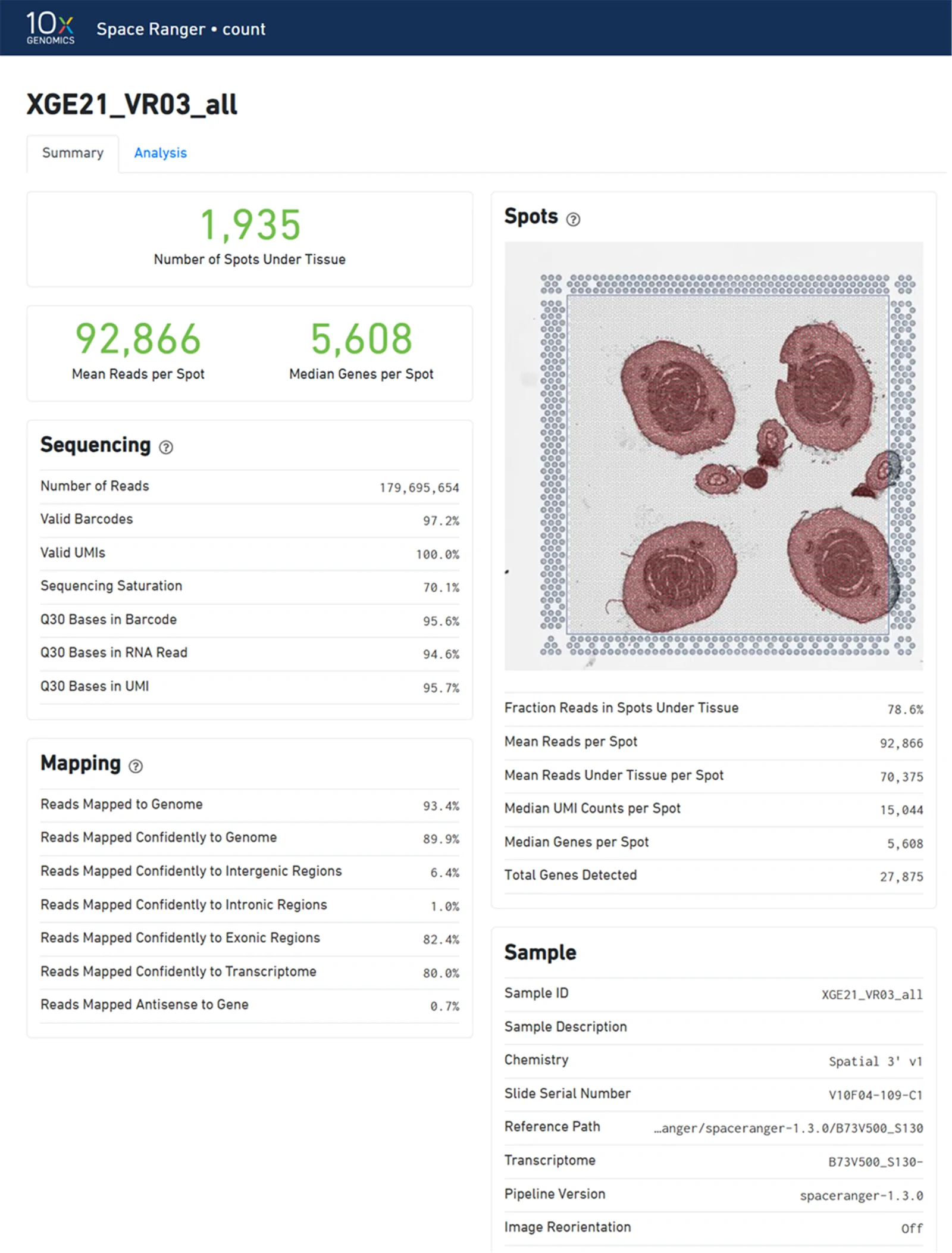
Representative Space Ranger web summary. The report shows tissue-covered spots and key sequencing, mapping, and gene detection metrics for sample XGE21_VR03. A total of 1,935 spots were identified under tissue, with 92,866 mean reads and 5,608 median genes per spot. The fraction of reads in tissue-covered spots was 78.6%.

**F2. Import Visium output into R and prepare spatial objects**

1. Import the Space Ranger output into R using Seurat (or STUtility).

2. Prepare an input table containing the following files for each capture area:

a. Filtered feature-barcode matrix, such as “filtered_feature_bc_matrix.h5”.

b. High-resolution tissue image, such as “tissue_hires_image.png”.

c. Scalefactor file, such as “scalefactors_json.json”.

d. Sample metadata, including sample ID, capture area ID, section number, and section order.

3. Use “load10x()” [or InputFromTable()] to generate the spatial object. The GitHub workflow gives an example using these Space Ranger output files as input.

4. Add metadata for biological replicates, capture area, section number, and anatomical domain.

*Notes:*

*1. The 14 .rds data objects and associated metadata from our study have been deposited in Zenodo (* *https://zenodo.org/records/22058284**). The GitHub repository is primarily designed for the Seurat v5 data structure. The STUtility dataset is intended for 3D reconstruction and visualization. Care should be taken when applying additional processing steps to the STUtility dataset while using the repository.*

*2. The raw feature-barcode matrix (.h5) file can be loaded to obtain the unmasked expression pattern, which allows checking the degree of transcript horizontal overflow.*

**F3. Perform quality control and filtering**

1. Merge the datasets from 14 capture areas (biological samples) and calculate quality-control metrics for each tissue-covered spot, including:

a. Number of detected genes.

b. Total UMI counts.

c. Percentage of mitochondrial gene reads.

d. Percentage of chloroplast gene reads.

e. Correlation between unique genes and read counts.

2. Remove genes with fewer than 100 total raw UMI counts across the dataset. Exclude spots in which mitochondrial or plastid-derived reads account for more than 5% of the total reads.

*Note: These QC thresholds serve as general guidelines; specific cutoffs should be adjusted based on the tissue type, sample quality, and biological context under study.*

3. Exclude tissue-covered spots that overlap two or more anatomical domains when performing domain-level analyses.

4. Visualize quality-control metrics using histograms, violin plots, and scatter plots. In the original study, mitochondrial gene reads were below 4%, and chloroplast gene reads were below 1% in most tissue-covered spots, supporting high data quality (Figure 10).

**Figure 10.**
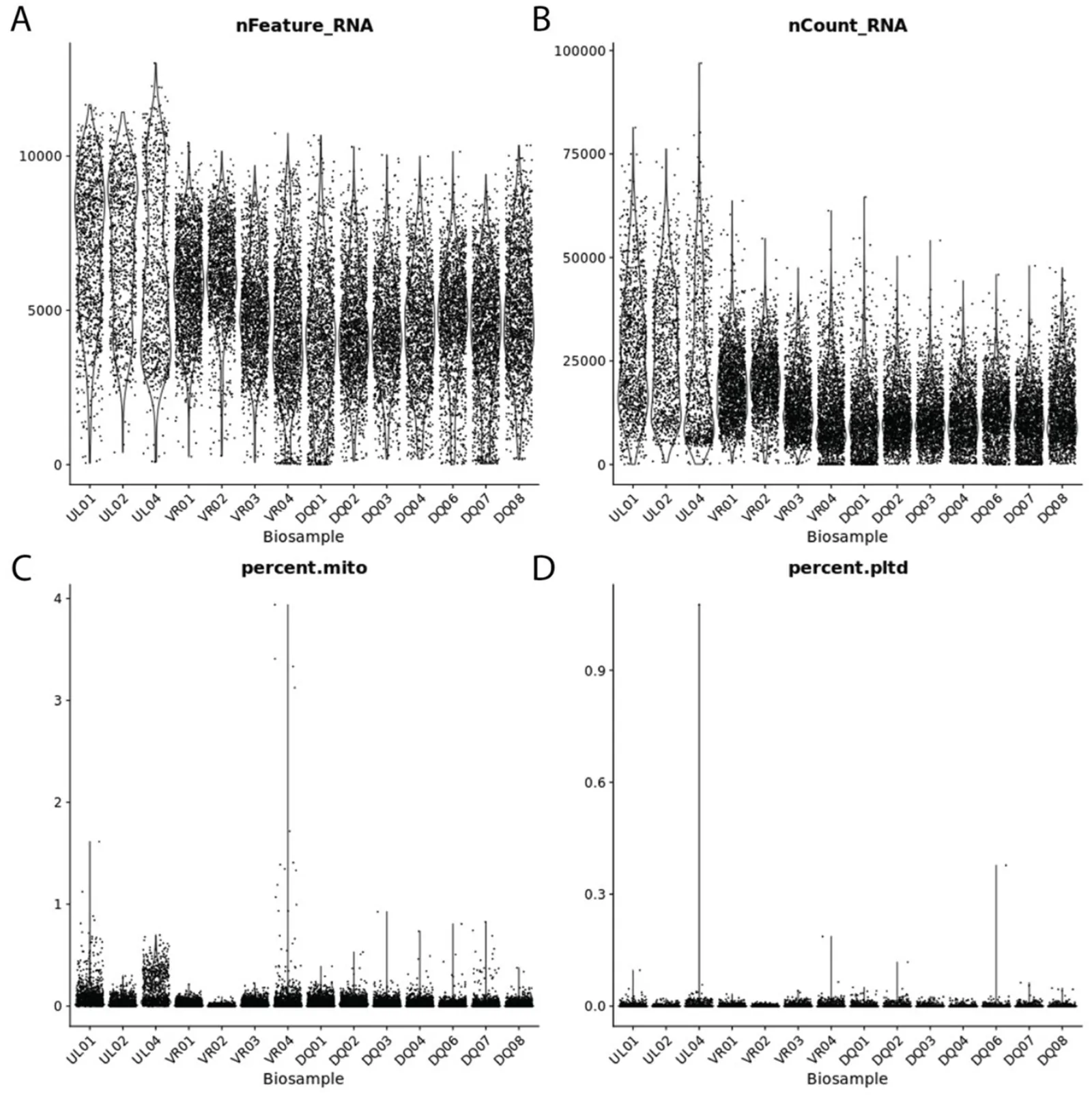
Distribution of quality-control metrics of representative spatial transcriptomics samples. Violin plots show (A) the number of detected genes (nFeature_RNA), (B) total UMI counts (nCount_RNA), (C) the percentage of mitochondrial transcripts, and (D) the percentage of chloroplast transcripts per tissue-covered spot. Each point represents an individual spot. Adapted from Supplementary Figure 7 of [1].

**F4. Normalize, integrate, and cluster Visium datasets**

1. Normalize the merged spatial transcriptomic dataset using SCTransform() with vst.flavor = "v2" and retain 3,000 variable features.

2. Perform PCA on the SCT assay. Calculate 50 PCs initially and inspect the elbow plot. In the demonstration dataset, retain PCs 1–30 for downstream analysis.

3. Integrate the sample-specific SCT layers using IntegrateLayers(method = HarmonyIntegration) (Figure 11).

4. Construct the nearest-neighbor and shared-nearest-neighbor graphs from the Harmony reduction.

5. Perform graph-based clustering at resolution 2.0 and generate a UMAP embedding from the Harmony reduction.

*Note: SCTransform() performs normalization and variance stabilization, whereas IntegrateLayers(method = HarmonyIntegration) integrates the sample-specific layers and reduces technical variation. Integration does not replace normalization. The number of PCs and clustering resolution should be reevaluated for new datasets.*

**Figure 11.**
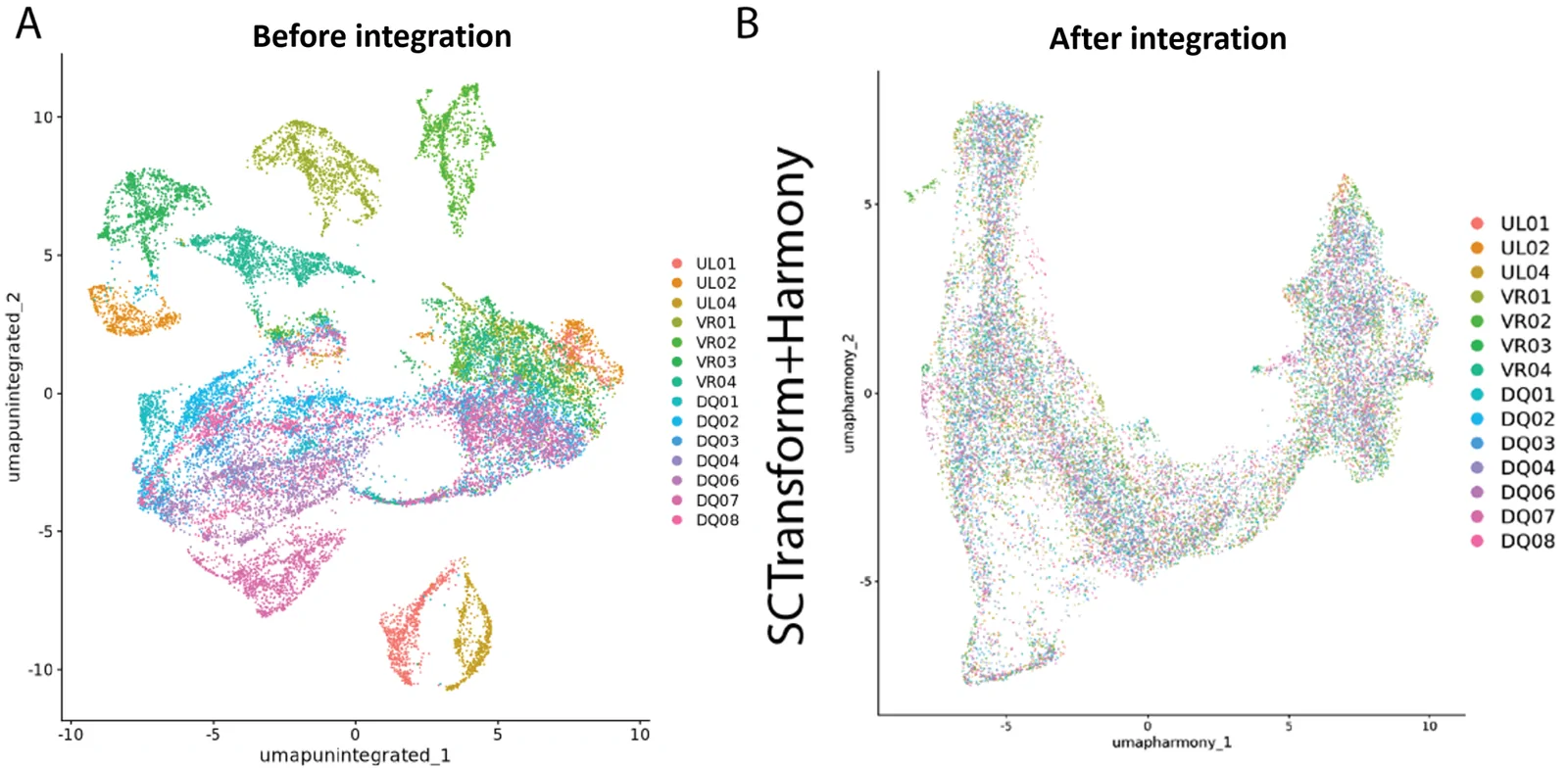
Effect of SCTransform normalization and Harmony integration. Uniform manifold approximation and projection (UMAP) embeddings of tissue spots from 14 biological samples, colored by sample identity. (A) Before integration, 14 biological samples form distinct groups, indicating sample-associated variation. (B) After SCTransform normalization and Harmony integration, spots from different biological samples are more evenly mixed, consistent with reduced batch effects. Adapted from Supplementary Figures 8A and 9A of [1].

**F5. Annotate structural domains**

1. Overlay tissue-covered spots onto the histological images.

2. Assign each spot to one of the structural domains based on anatomical position: SAM, P1_P2, P3, P4, P5, coleoptile, or coleoptile vein, co_v.

*Note: Manual annotation can be performed using 10x Loupe Browser to define custom clusters, which can then be reintegrated into the Seurat object.*

3. Group P1 and P2 together as P1_P2 when their signals cannot be separated because of the 55-μm spot diameter.

4. Exclude spots covering two or more structural domains from domain-level analysis.

**Critical:** Domain annotation should use both histological information and gene expression patterns. Clustering alone is not sufficient because neighboring developmental domains can have gradual transcriptional transitions.

**F6. Identify marker genes and tissue supergroups**

1. Use the integrated Seurat object to perform unsupervised clustering.

2. Identify cluster marker genes using “FindAllMarkers()”.

3. Compare the clusters and candidate markers with histological images and structural-domain labels.

4. Group transcriptionally and anatomically related clusters into tissue supergroups. In the original study, unsupervised clustering identified 33 clusters, which were then classified into 12 major supergroups based on anatomical correspondence and gene expression patterns (Figure 12).

**Figure 12.**
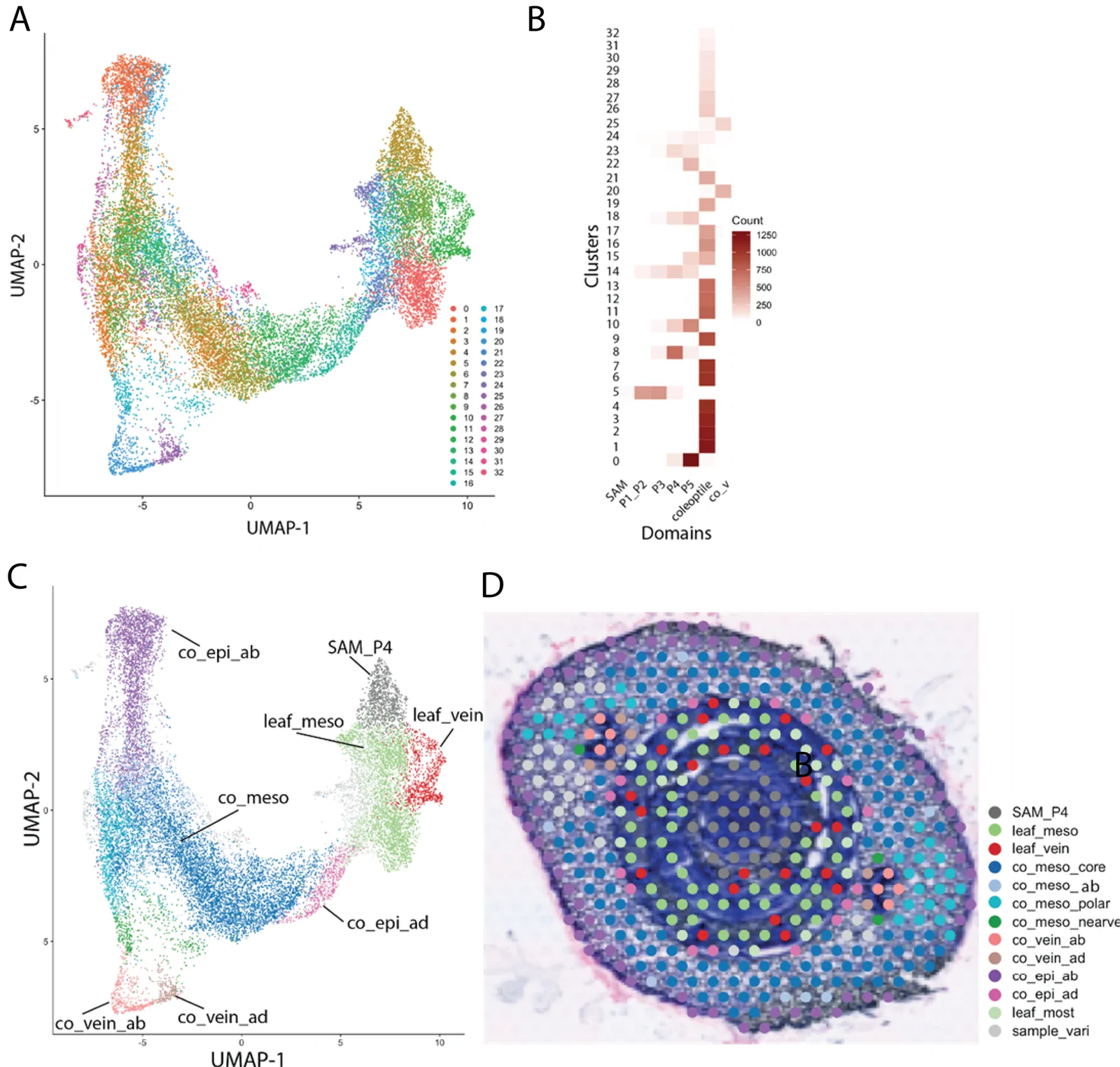
Unsupervised clustering and anatomical identification of maize shoot tissue supergroups. (A) Uniform manifold approximation and projection (UMAP) of tissue-covered spots after SCTransform normalization and Harmony integration, colored by 33 unsupervised clusters. (B) Distribution of the clusters across seven structural domains. Color intensity indicates the number of spots in each cluster–domain combination. (C) UMAP showing the grouping of clusters into 12 tissue supergroups based on transcriptional similarity and anatomical correspondence. The sample_vari group represents sample-specific variation. (D) Spatial mapping of the tissue supergroups onto a representative histological section; colors correspond to those in panel C. Abbreviations: co, coleoptile; epi, epidermis; meso, mesophyll; ad, adaxial; ab, abaxial; co_v, coleoptile vein. Adapted from Figure 4A, B and Supplementary Figure 13A, D of [1].

5. Define differentially expressed genes using the following criteria:

a. Log2 fold change > 1 or < -1.

b. Adjusted p-value < 0.05.

*Note: The cutoffs above represent baseline filtering criteria. Researchers should calibrate these parameters according to sample depth, baseline background noise, and the specific target effect size.*

**F7. Generate pseudobulk profiles for structural domains**

1. Treat each individual seedling, identified by sample_id, as an independent biological replicate (n = 14). Sum raw RNA UMI counts across all retained spots belonging to the same biological replicate and structural domain to generate one pseudobulk library for each replicate–domain combination. Spots and serial sections from the same seedling are treated as subsamples, not independent replicates.

2. Normalize the replicate–domain pseudobulk libraries jointly using the trimmed mean of M-values (TMM) method in edgeR and transform the normalized expression values to log_2_ counts per million using a prior count of 1. Generate profiles for SAM, P1_P2, P3, P4, P5, coleoptile, and co_v.

3. Use the replicate-level pseudobulk profiles to evaluate variation among biological replicates. For descriptive visualization, calculate the unweighted mean log_2_-CPM profile across biological replicates for each structural domain.

4. Perform PCA on the seven domain-mean profiles using centered but unscaled gene expression values. Perform hierarchical clustering of the same profiles using Euclidean distance and complete linkage. These analyses describe gene expression similarities among structural domains and are not used for inferential testing.

In the original study, pseudobulk PCA separated SAM, developing leaves, coleoptile, and coleoptile vein into major groups. Hierarchical clustering showed that coleoptile was the most distinct, followed by co_v, whereas SAM and P1_P2 clustered closely, and P3/P4 formed another close subcluster (Figure 13).

**Figure 13.**
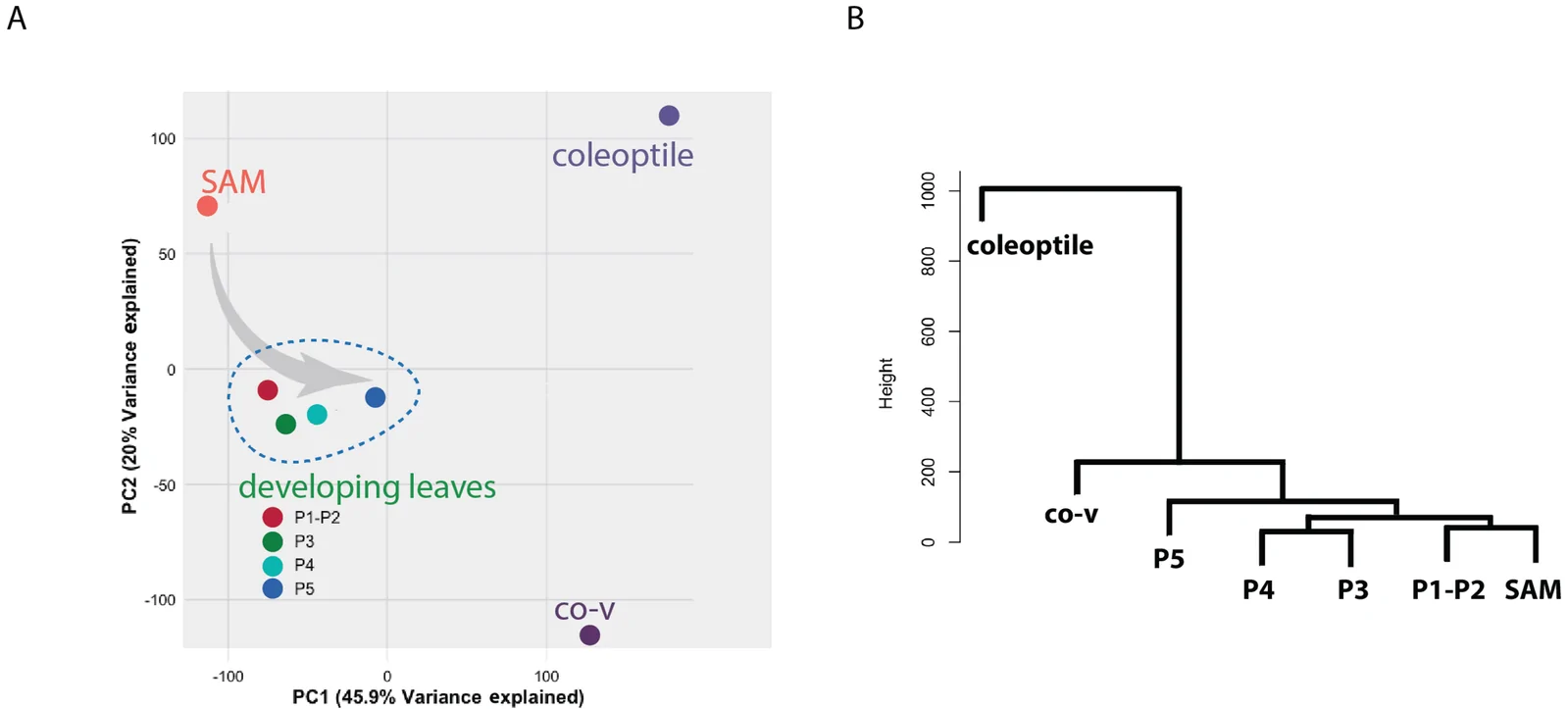
Pseudobulk gene expression profiles of maize shoot domains. (A) PCA showing that developing leaf primordia (P1–P5) cluster together, whereas the shoot apical meristem (SAM), coleoptile, and coleoptile vein (co_v) are more distinct. The gray arrow indicates the proposed developmental progression. (B) Hierarchical clustering of the same domains; branch height represents gene expression dissimilarity. Adapted from Figure 3A, B of [1].

**F8. Analyze developmental gene expression trends and GO enrichment**

1. Extract normalized expression values from the data layer of the SCT assay. Within each biological replicate, average spot-level expression values for each structural domain to generate replicate–domain profiles.

2. Calculate the mean unscaled expression profile for each domain and retain genes with non-zero mean expression in all seven domains. In the demonstration analysis, intersect these genes with the predefined gene list in sub_gene.csv.

3. Standardize each gene across the replicate–domain profiles by subtracting its mean and dividing by its standard deviation.

4. For each gene, calculate the mean scaled expression across biological replicates within each structural domain.

5. Cluster the domain-level gene expression profiles using Euclidean distance and complete-linkage hierarchical clustering.

6. Cut the dendrogram into seven gene expression trend clusters. Seven clusters were empirically selected in the original dataset because this cutoff provided an interpretable summary of the major developmental expression patterns.

*Note: Users should inspect the dendrogram and biological interpretability before applying this cutoff to other datasets.*

7. Within each cluster, rank genes by their mean SCT expression and retain up to 1,000 genes for GO enrichment analysis. The 1,000-gene limit is a predefined pragmatic cutoff rather than a statistically optimized threshold.

*Note: This value was arbitrarily defined.*

8. Perform GO enrichment using agriGo or an equivalent hypergeometric enrichment method with an explicitly defined background gene set. Apply an appropriate multiple-testing correction when interpreting newly calculated enrichment results. In the original study, Clusters 1–3 showed high expression in SAM, Clusters 4–6 showed gradual changes across leaf primordia, and Cluster 7 showed high expression in coleoptile and co_v. GO analysis associated Cluster 1 with gene expression, metabolism, development, and cell-cycle processes, Cluster 6 with organelle organization and chromatin modification, and Cluster 7 with defense, stress responses, and secondary metabolism (Figure 14).

**Figure 14.**
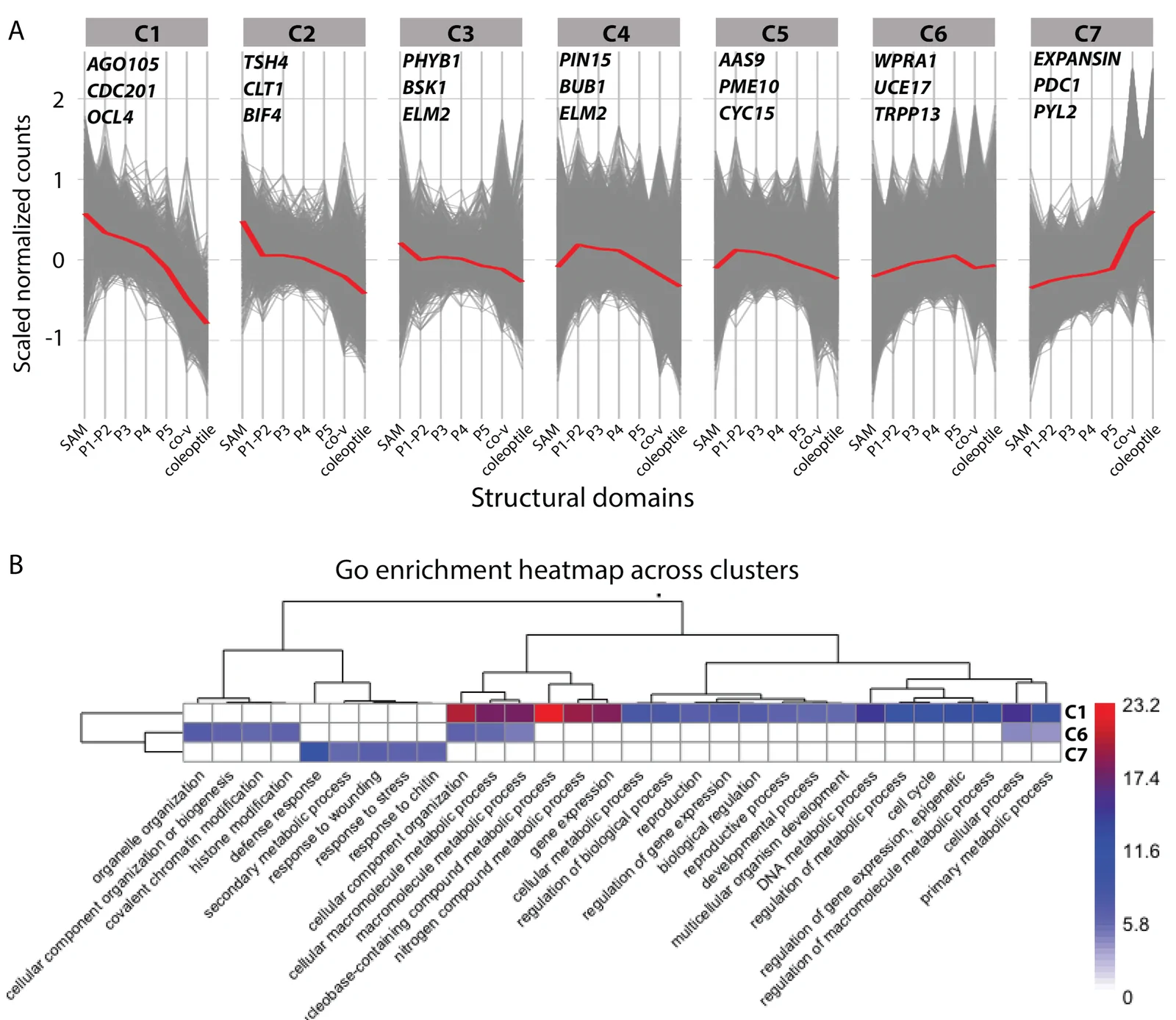
Gene expression profiles of developing maize shoot. (A) Seven hierarchical clusters of gene expression patterns. Gray lines represent individual genes, and the red line indicates the median expression trend. Three representative genes are shown for each cluster. (B) GO enrichment heatmap for Clusters 1, 6, and 7 (p < 1 × 10^-6^). Colors represent -log_10_(p). Adapted from Figure 3C, D of [1].

**F9. Perform RNA velocity analysis of the embryonic leaf**

1. For each of the 14 selected Space Ranger libraries, corresponding to 14 biological replicates, process the mapped BAM file using Velocyto.py v0.17.17 and the Zm-B73-Reference-NAM-5.0 gene annotation. Generate one loom file per library containing spliced, unspliced, and ambiguous UMI-count layers.

2. Export the raw RNA-count matrix, spot metadata, PCA coordinates, and umap.harmony coordinates from XGE202122_S5_subset_embleaf_harmony_join.rds. Define biological_replicate from the sample metadata field.

3. Import the 14 selected loom files into Python and verify that every file contains the required spliced and unspliced layers. The ambiguous layer may be retained, but is not used for velocity estimation.

4. Convert the loom barcodes to the corresponding Seurat spot identifiers and match spots using exact barcode identities. Retain only spots assigned unambiguously to SAM, P1_P2, P3, P4, or P5, thereby excluding spots spanning multiple structural domains and spots assigned to coleoptile or co_v.

5. Transfer the biological replicate, section, structural domain, PCA, and UMAP information from the Seurat export to the corresponding loom-derived observations.

6. Combine the 14 annotated loom-derived objects into one AnnData object, retaining genes shared among the selected loom files and the Seurat RNA-count matrix. In the example dataset, this procedure retained 6,392 spots and 39,756 shared genes.

7. Filter genes using min_shared_counts = 20, normalize counts per spot, apply log transformation, and retain the top 2,000 highly variable genes. Calculate first- and second-order moments using the first 30 Seurat-derived principal components and 30 nearest neighbors.

8. For a controlled model comparison, first calculate stochastic velocity using velocity(mode = "stochastic"). Next, estimate gene-specific kinetic parameters using recover_dynamics(max_iter = 20) and calculate dynamical velocity using velocity(mode = "dynamical"). Store the stochastic and dynamical results under separate velocity keys so that their layers and directed graphs are not overwritten. The dynamical model is used as the primary analysis.

9. Calculate a separate velocity graph for each model and plot velocity vectors and streamlines on the transferred umap.harmony embedding. Display the dynamical result in Figure 15B and use the stochastic result as a sensitivity comparison.

The analysis retains individual spots as observations; it does not aggregate expression by biological replicate. Biological-replicate identifiers are used for loom assignment and quality-control summaries. In this dataset, unspliced UMIs accounted for approximately 7% of the combined spliced and unspliced UMI counts. Despite this relatively small unspliced fraction, the inferred velocity field was broadly consistent with the principal SAM-to-P5 developmental progression. Because Visium spots contain mixtures of cells, these vectors represent predicted changes in spot-level transcriptional states rather than literal single-cell movement or lineage.

**F10. Perform pseudotime analysis using Monocle 3**

1. Load the 14 individual Seurat objects and retain spots assigned unambiguously to SAM, P1_P2, P3, P4, or P5. Retain spots with at least 100 total UMIs and convert each filtered Seurat object into a Monocle 3 cell_data_set.

2. Combine the 14 cell_data_set objects using combine_cds(keep_all_genes = TRUE), preserving the globally unique spot barcodes and curated metadata.

3. Preprocess the combined dataset using preprocess_cds(method = "PCA", norm_method = "log", num_dim = 100). Here, norm_method = "log" specifies log normalization, whereas num_dim = 100 specifies the number of principal components retained.

4. Use align_cds(preprocess_method = "PCA", alignment_group = "section_id") to reduce section-associated technical variation.

5. Transfer the umap.harmony coordinates from the processed previous rds object (used for RNA velocity analysis in F9) to the combined Monocle 3 object. For a new dataset without reference coordinates, calculate a new UMAP from the section-aligned PCA representation.

6. Cluster the spots and learn a single tree-like principal graph using cluster_cells() and learn_graph(use_partition = FALSE, close_loop = FALSE).

7. Open the interactive Monocle 3 root-selection interface and manually select one or more principal graph nodes located in the SAM region. Save the selected node identifiers so that the same roots can be reused in subsequent non-interactive analyses.

8. Estimate pseudotime using order_cells() with the selected SAM-associated root nodes and visualize pseudotime and the inferred principal graph on the transferred UMAP coordinates (Figure 15C).

*Note: The inferred pseudotime direction depends on the biologically informed selection of the SAM root. Root selection should therefore be reported explicitly and reevaluated when the workflow is applied to another dataset.*

**Figure 15.**
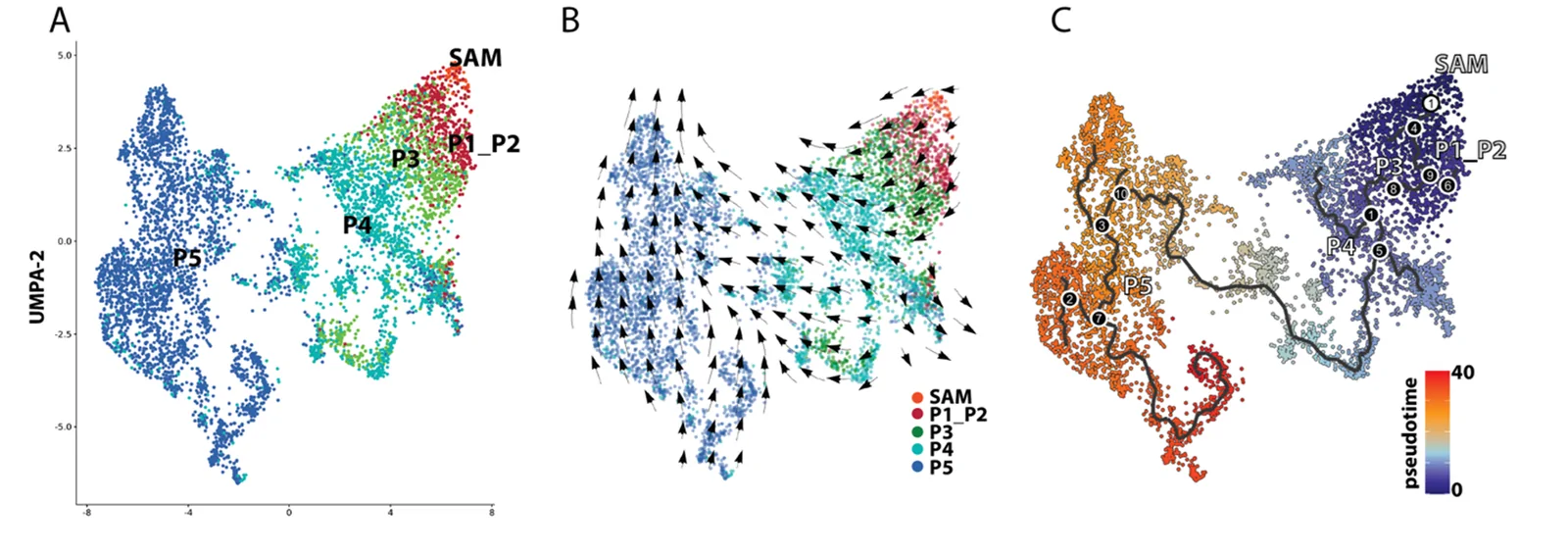
Developmental trajectory associated with dynamic gene expression profiles of the shoot apical meristem (SAM) and embryonic leaves of shoots. (A) Uniform manifold approximation and projection (UMAP) maps of the maize shoot spatial transcriptomes showing expression dynamics of tissue spots designated by developmental features of embryonic leaves. (B) RNA velocity analysis. (C) The pseudotemporal ordering of colored spots of structural domains. Adapted from Figure 5A–C of [1].

**F11. Construct time-ordered transcription factor co-expression networks**

1. Prepare a list of maize transcription factor genes using the collected transcription factor datasets.

2. Calculate mean expression values for each structural domain using “AverageExpression()”.

3. Normalize UMI counts by the total number of tissue spots in each structural domain.

4. Apply TO-GCN to assign transcription factor genes to upregulation time-order levels. See the developer’s GitHub repository for guidance [10].

5. Identify co-expressed genes associated with transcription factors in each TO-GCN level.

6. Perform functional enrichment analysis using both transcription factors and co-expressed genes in each level.

7. Use all expressed genes as the background set.

8. Perform enrichment using Fisher’s exact test with FDR < 0.05.

*Note: Perform functional-enrichment analysis using Fisher’s exact test and consider functional categories with an FDR-adjusted p-value < 0.05 to be significantly enriched. Because many functional categories are tested simultaneously, FDR correction is applied to control for multiple hypothesis testing. The cutoff of 0.05 limits the expected proportion of false discoveries among the significant results while retaining sufficient sensitivity to identify biologically relevant functional categories. Users may apply a more stringent threshold when analyzing a very large number of categories or when a stronger control of false-positive results is required.*

**F12. Integrate the Visium dataset with maize scRNA-seq data**

1. Download the maize shoot scRNA-seq datasets used as the reference. In the original study, SRA runs SRR11943512 and SRR11943513 were used. Quantify the sequencing reads and prepare one 10x-formatted gene-count matrix for each library.

2. Import each raw gene-count matrix into Seurat using CreateSeuratObject(min.cells = 3, min.features = 200).

3. Run scDblFinder separately for each library and retain cells classified as singlets.

4. Prefix cell barcodes with the corresponding library identifier and merge the two singlet objects while retaining separate RNA count layers.

5. Normalize the layered reference object using SCTransform v2 with method = "glmGamPoi" and retain 3,000 variable features.

6. Calculate 50 principal components for inspection of the PCA elbow plot. In the demonstration dataset, the contribution of additional components decreased substantially after approximately 30 PCs; therefore, recalculate and retain the first 30 PCs for downstream integration.

*Note: Users should inspect the elbow plot and adjust this number according to the size and complexity of their datasets.*

7. Integrate the two libraries using “IntegrateLayers(method = HarmonyIntegration)”.

8. Join the RNA layers using JoinLayers() after integration. Join the SCT layers as well if multiple SCT count or data layers remain.

9. Construct the nearest-neighbor and shared-nearest-neighbor graphs using dimensions 1–30 of the Harmony reduction. Perform graph-based clustering at resolution 2.0 and calculate the Harmony UMAP using the same dimensions. Set the random seed to 2026.

*Note: Clustering resolution controls cluster granularity. A resolution of 2.0 was selected to retain transcriptionally distinct and relatively uncommon cell populations in the demonstration dataset. Users should test multiple resolutions and evaluate cluster stability, marker expression, and biological interpretability.*

10. Annotate the integrated scRNA-seq reference using curated maize marker genes and SCINA. Remove markers shared by multiple cell types, retain up to 100 ranked markers per type, and omit cell types represented by fewer than two usable markers. Run SCINA using max_iter = 100, convergence_n = 10, sensitivity_cutoff = 1, rm_overlap = TRUE, and allow_unknown = TRUE. Label cells with a maximum posterior probability below 0.5 as Unknown.

11. Validate the resulting cell-type assignments by examining marker expression and cell-type distributions on the Harmony UMAP.

**F13. Assign cell identities using SCINA**

1. Prepare a marker table containing the required columns gene_id and cell_type. Optionally, include avg_log2FC, avg_logFC, marker_rank, or rank to define marker priority.

2. Intersect the marker genes with the genes present in the SCT assay of the integrated scRNA-seq reference.

3. Remove genes assigned as markers for more than one cell type.

4. Rank the retained markers within each cell type. When a signature contains more than 100 genes, retain the 100 highest-ranked genes. If no ranking column is available, retain genes according to their order in the input marker table.

5. Remove cell types represented by fewer than two remaining marker genes.

6. Convert the filtered marker table into a named list containing one gene vector per cell type.

7. Extract the normalized data layer of the SCT assay for the retained marker genes.

8. Set the R random seed to 1 and run SCINA v1.2.0 using:

a. “max_iter = 100”

b. “convergence_n = 10”

c. “sensitivity_cutoff = 1”

d. “rm_overlap = TRUE”

e. “allow_unknown = TRUE”

*Note: allow_unknown = TRUE permits cells that are insufficiently supported by the supplied signatures to remain unassigned instead of forcing them into a known cell type. Although overlapping markers are removed explicitly during signature preparation, rm_overlap = TRUE provides an additional safeguard within SCINA.*

9. Assign each cell the label with the highest posterior probability.

10. Label cells as Unknown when their maximum posterior probability is below 0.5 or is not finite.

*Note: The posterior threshold of 0.5 is a predefined baseline rather than a universally optimal cutoff. Users should examine posterior distributions, marker expression, and biological plausibility when calibrating this threshold for another dataset.*

11. Add the SCINA label and maximum posterior probability to the Seurat metadata while preserving the existing active identities.

12. Validate the annotations by highlighting each assigned cell type on the Harmony UMAP and examining the expression of established marker genes (Figure 16).

**Figure 16.**
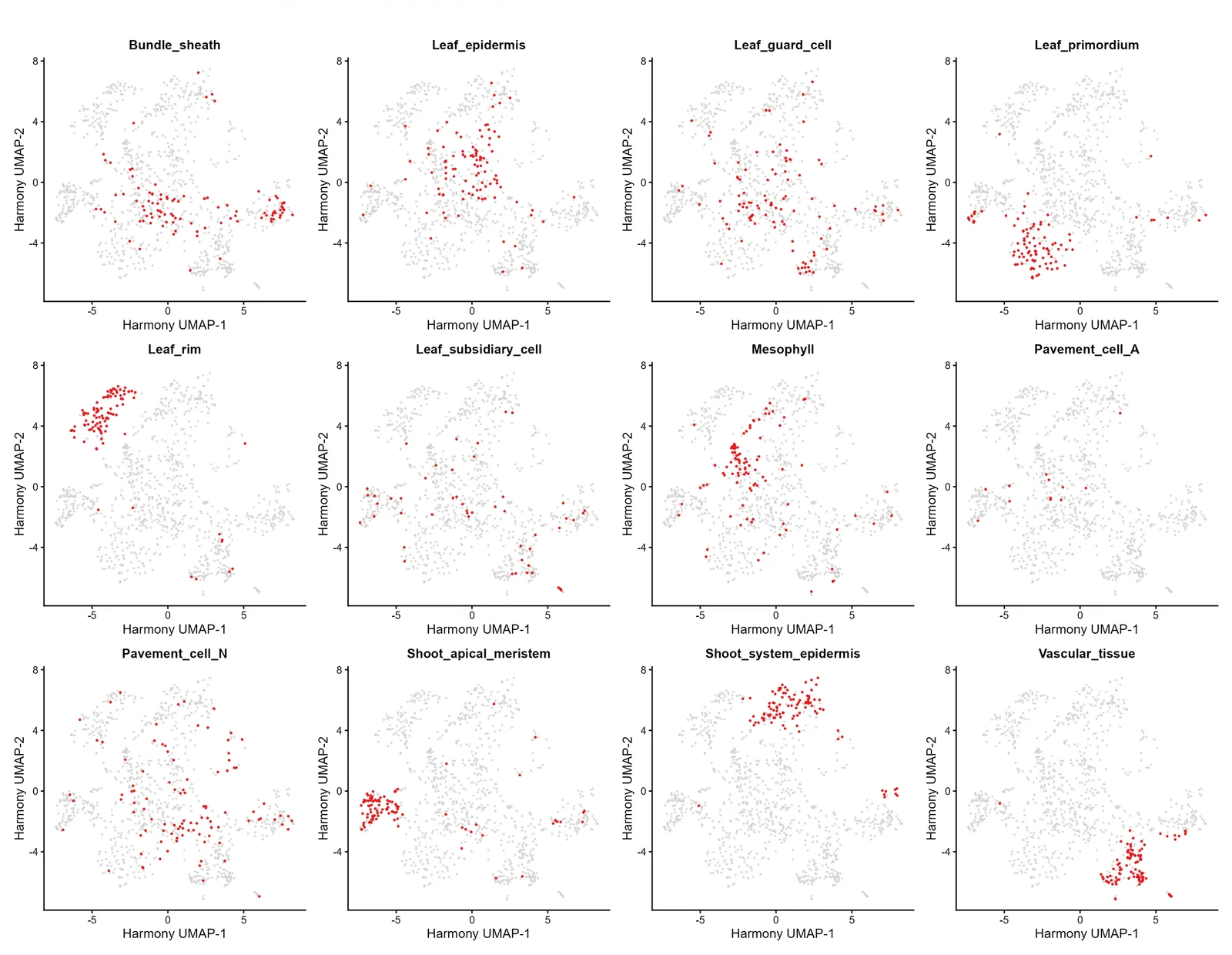
Harmony-corrected uniform manifold approximation and projection (UMAP) overlays validating the SCINA cell-type assignments. Each panel highlights one annotated cell type in red, while all other cells are shown in gray. The displayed categories include shoot system epidermis, pavement cells, leaf epidermis, mesophyll, leaf rim, shoot apical meristem, vascular tissue, bundle sheath, leaf primordium, guard cells, and subsidiary cells when present in the analyzed object.

**F14. Map scRNA-seq cell-type information onto Visium spots**

1. Use the SCINA-annotated maize scRNA-seq object as the reference and the processed embryonic-leaf Visium object as the query. In the demonstration analysis, retain the 6,392 Visium spots assigned unambiguously to SAM, P1_P2, P3, P4, or P5.

2. Remove cells labeled Unknown from the scRNA-seq mapping reference.

3. Perform hard cell-type label transfer using SCT-normalized reference and query assays. Identify transfer anchors using “FindTransferAnchors()”.

4. Transfer the reference cell-type labels and prediction scores to the Visium spots using MapQuery(). Add the predicted labels and scores to the Visium metadata.

5. Visualize the transferred labels on the existing umap.harmony coordinates of the embryonic-leaf Visium object. These coordinates are retained to maintain consistency with the other Visium analyses.

6. For soft deconvolution, harmonize the gene identifiers between the annotated scRNA-seq reference and the Visium query. Exclude mitochondrial, plastid, and ribosomal genes, select approximately 3,000 highly variable genes, and retain up to 100 positively enriched marker genes per reference cell type based on AUC and log fold change.

7. Downsample the scRNA-seq reference to at most 100 cells per cell type and run SPOTlight separately for each physical Visium section using raw scRNA-seq and Visium UMI counts, min_prop = 0.01, and an R random seed of 1.

8. Normalize each estimated spot-level proportion vector to sum to 1 and add the resulting proportions to the Visium metadata using column names beginning with SPOT_.

9. Visualize the continuous cell-type proportions on the Visium Harmony UMAP and on the corresponding tissue sections. Use spatial scatter-pie plots to display mixed cell-type compositions within individual spots.

10. For visualization of selected enriched spots, apply cell type–specific empirical thresholds. In the original analysis, vascular-enriched spots were displayed using SPOT_Vascular_tissue > 0.10, whereas SAM-enriched spots were displayed using SPOT_Shoot_apical_meristem > 0.05.

*Note: These values are empirical visualization thresholds from the original dataset and are not statistical significance cutoffs. For another dataset, thresholds should be evaluated using known marker expression, anatomically appropriate positive and negative regions, and sensitivity analyses across multiple candidate cutoffs.*

11. As reliability checks, compare the maximum-proportion SPOTlight cell type with the Seurat-transferred hard label and, when independent marker sets are available, calculate correlations between SPOTlight proportions and cell-type module scores (Figure 17).

**Figure 17.**
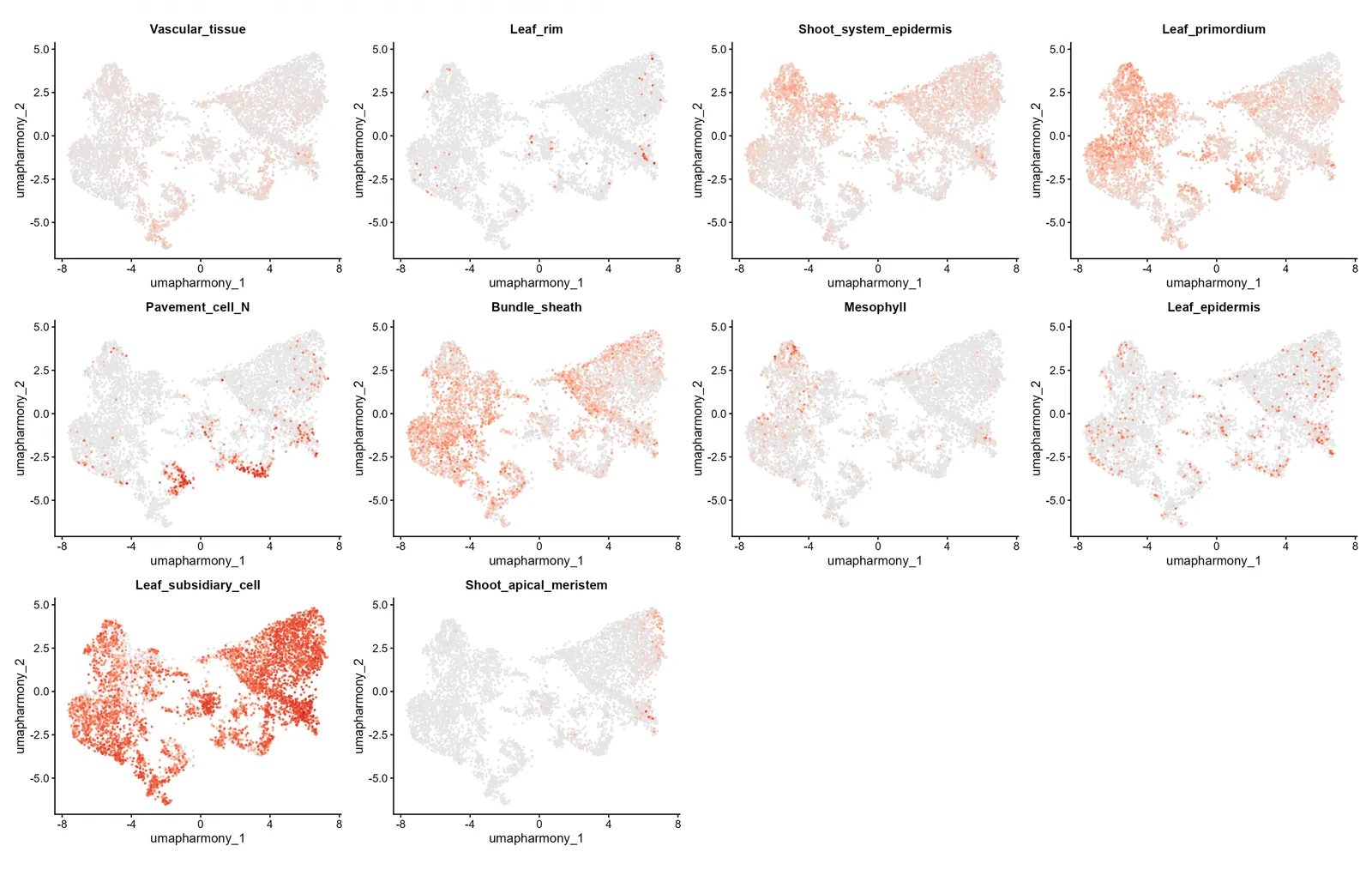
SPOTlight-estimated cell-type proportions on the embryonic-leaf Harmony UMAP. Estimated proportions of 10 cell types were projected onto the Harmony UMAP of Visium spots from the SAM–P5 dataset. Each point represents one Visium spot, and color intensity indicates the SPOTlight-estimated proportion of the indicated cell type, ranging from low or absent (light gray) to high (red). Cell types shown are vascular tissue, leaf rim, shoot-system epidermis, leaf primordium, pavement cell N, bundle sheath, mesophyll, leaf epidermis, leaf subsidiary cell, and shoot apical meristem.

**F15. Reconstruct serial sections in 3D**

1. Split each capture area into separate section datasets using the STUtility R package [11].

2. Modify the histological image for each dataset so that only one section is retained.

3. Assign each section a section index and z-position according to the physical order of sectioning.

4. Use “ManualAnnotation()” in STUtility to match tissue-covered spots to the corresponding section image.

5. Use the modified STUtility 3D workflow to reconstruct spatial-temporal gene expression profiles.

6. Inspect candidate genes and marker genes in 3D.

7. For an alternative interactive visualization, use the Napari-based workflow [12]:

a. Expand each spot by a user-defined radius.

b. Generate binary masks.

c. Apply connected-component labeling.

d. Register regions to a common reference using translational and rotational alignment.

e. Stack aligned slices along the z-axis.

f. Render gene expression as color-coded markers in the same coordinate space as the tissue morphology.

In the original study, *NAC119* and *HB46* were visualized across four serial sections of samples, and their 3D expression patterns were reconstructed using STUtility [11] (Videos 2 and 3) or Napari [12].

**Critical:** 3D reconstruction requires correct section order, accurate cropping, and reliable alignment. Misalignment can lead to incorrect interpretation of spatial expression gradients.

**Video 2.**
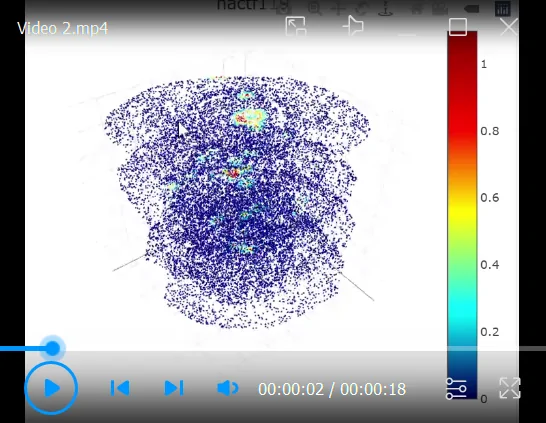
Three-dimensional expression patterns of *NAC119*. Expression across four serial maize shoot sections was aligned and reconstructed in 3D. Colors indicate normalized expression levels, from low (blue) to high (red). Originally published as Supplementary Video 1 in [1].

**Video 3.**
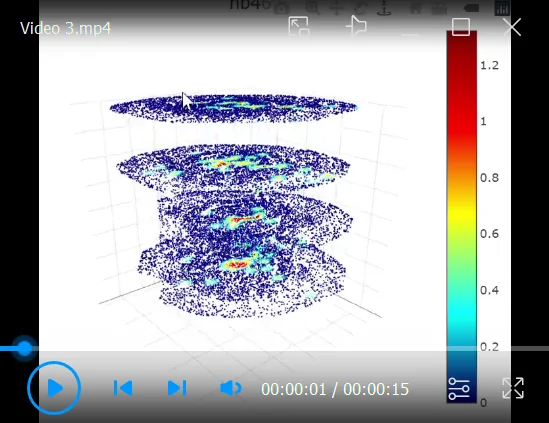
Three-dimensional expression patterns of *HB46*. Expression across four serial maize shoot sections was aligned and reconstructed in 3D. Colors indicate normalized expression levels, from low (blue) to high (red). Originally published as Supplementary Video 2 in [1].

## Validation of protocol

This protocol has been used and validated in the following research article:

• Wu et al. [1]. Serial Spatial Transcriptomes Reveal Regulatory Transitions in Maize Leaf Development. *Plant Biotechnology Journal*.

Application to small plant tissues: This section-positioning and cryosectioning approach was further tested using small and delicate plant samples, including the first true leaves of 6-DAI *Arabidopsis* seedlings and *Marchantia*. For both sample types, multiple cryosections from multiple specimens were successfully placed within a single capture area, demonstrating that the approach is suitable for arranging small plant tissues on spatial transcriptomics slides (Figure 18). For *Arabidopsis*, the first true leaf sections were positioned across the capture area and retained recognizable leaf anatomy after sectioning and imaging. For *Marchantia*, thallus sections were similarly arranged within the capture area, and anatomical structures were visible in the tissue images. cDNA fragment analysis from both sample types showed successful cDNA synthesis and amplification, supporting the compatibility of this sample preparation workflow with downstream 10x Visium library preparation (Figure 19). The Space Ranger summary showed satisfactory overall sequencing, mapping, and tissue-detection metrics (Figure 20).

**Figure 18.**
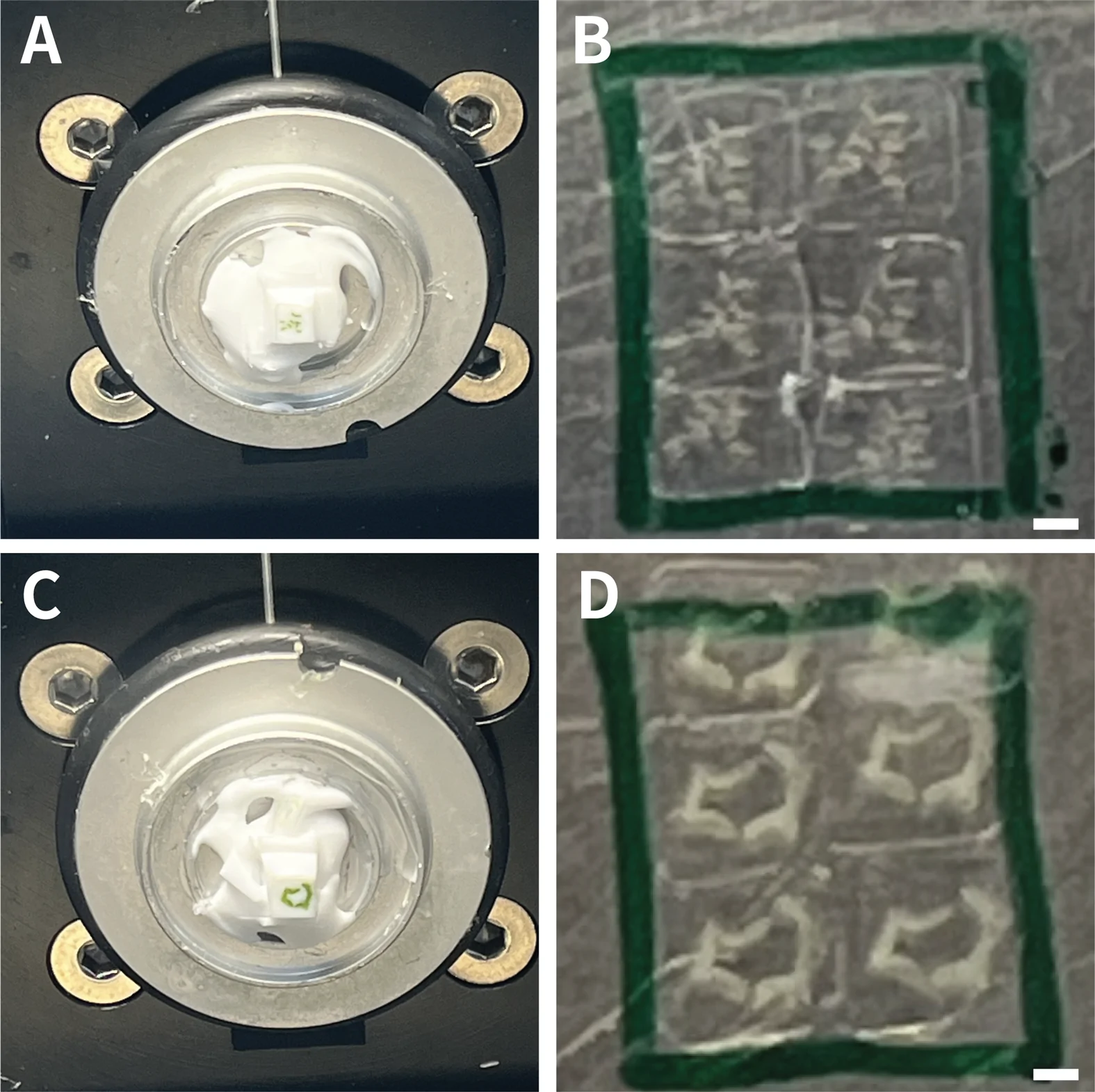
OCT embedding and cryosection placement for plant samples. Representative samples were embedded in OCT and oriented in the cryostat specimen holder before cryosectioning. Multiple *Arabidopsis* first true leaf specimens embedded in OCT are shown before sectioning (A), and multiple leaf sections were collected within the marked target area on the sample slide (B). *Marchantia* thalli embedded in OCT are shown before sectioning (C), and multiple thallus sections are similarly placed within the marked target area on the sample slide (D). The green outline indicates the region used to guide section placement and to ensure that sections are positioned within the target capture area for downstream spatial transcriptomic analysis. Scale bars, 1 mm.

**Figure 19.**
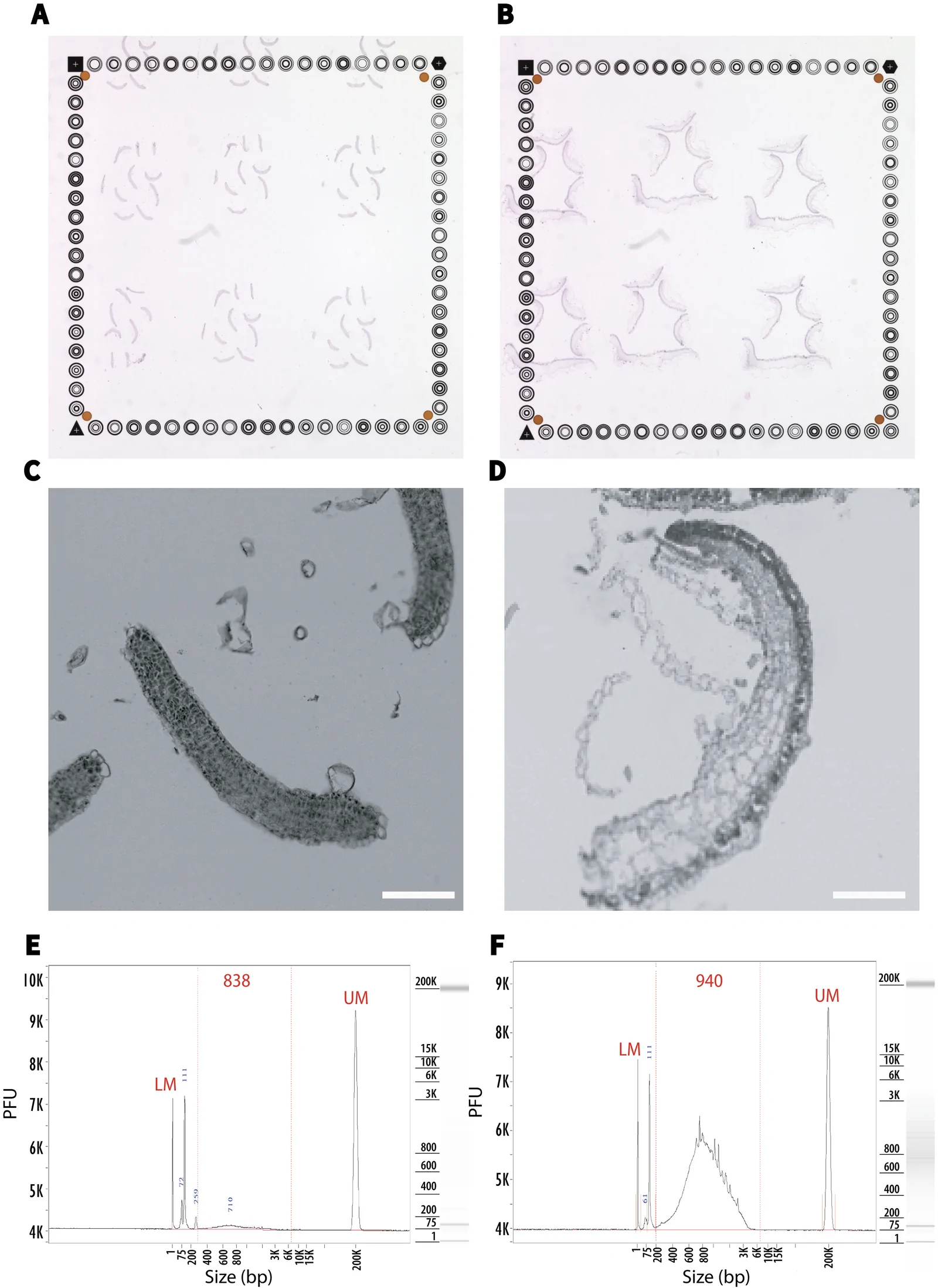
Application of the section-positioning workflow to small plant samples. (A, C, E) First true leaf sections from *Arabidopsis* seedlings at 6 days after imbibition (DAI). (B, D, F) *Marchantia* thallus sections. (A, B) Representative capture-area images showing multiple cryosections placed within the fiducial-marked capture area of a Visium HD 3’ Gene Expression slide. Tissue coverage, estimated by 10x Loupe Browser, is 5% for *Arabidopsis* (A), and 16% for *Marchantia* (B). (C, D) Representative anatomical images of cryosections. (E, F) Representative cDNA fragment analysis profiles after Visium reverse transcription. LM, lower marker; UM, upper marker. Scale bars, 100 μm.

**Figure 20.**
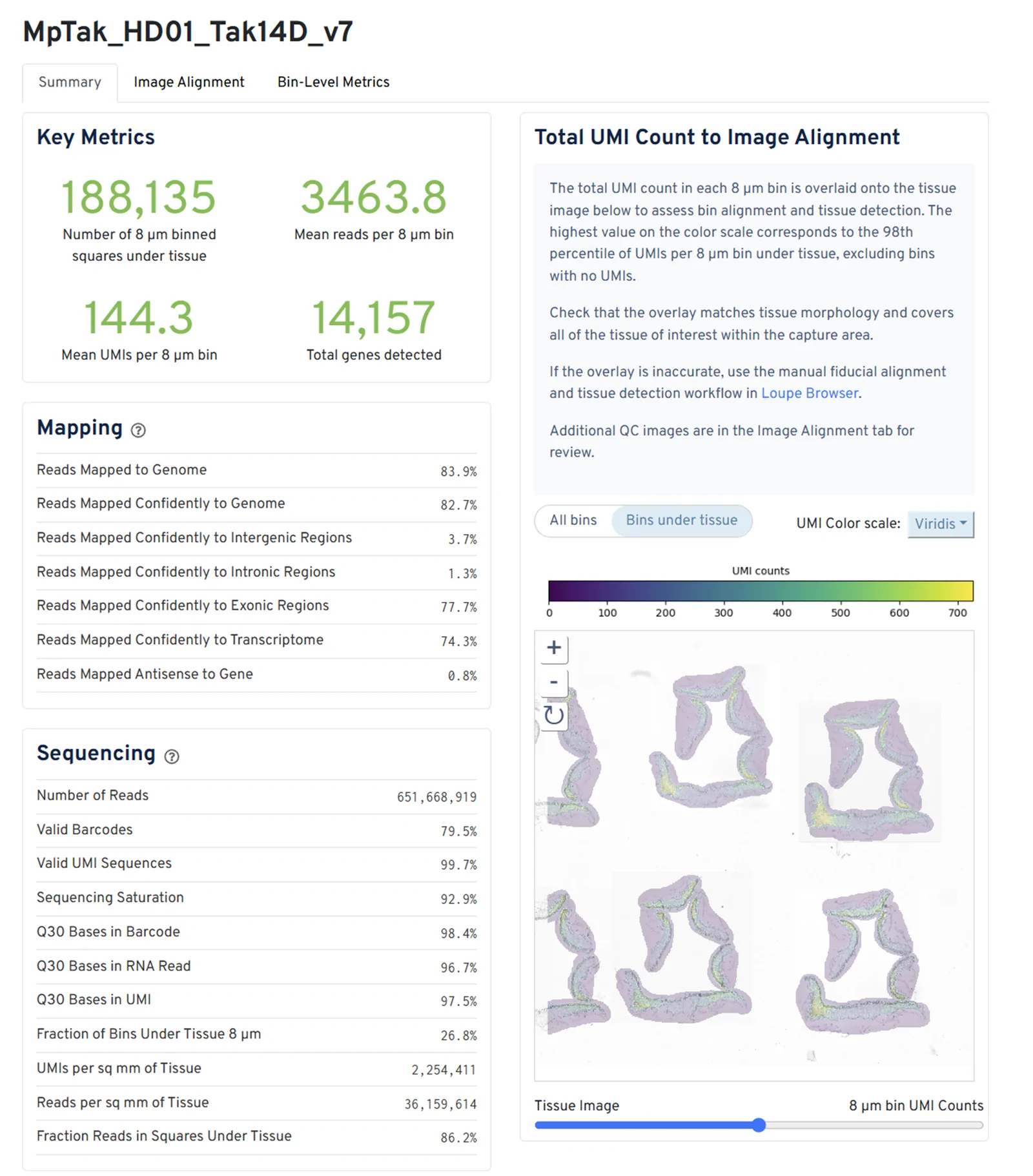
Representative Space Ranger web summary for a Visium HD 3’ dataset. The sample (MpTak_HD01_Tak14D_v7) was summarized at 8-μm bin resolution, yielding 188,135 bins under tissue, a mean of 3,463.8 reads and 144.3 UMIs per bin, and 14,157 detected genes. Sequencing saturation was 92.9%, and 86.2% of reads were assigned to bins under tissue. The right panel shows total UMI counts overlaid on the tissue image to assess tissue detection and image alignment.

## General notes and troubleshooting

**General notes**

1. This protocol was optimized using maize seedling shoot apices as the main example, but the same general strategy can be adapted to other small plant samples, including *Arabidopsis* first true leaves and *Marchantia* thalli. Sample size, tissue rigidity, air content, and sectioning behavior should be considered when adapting the protocol to other species or organs.

2. The size of the custom OCT embedding mold should be adjusted according to the sample size. In general, the mold should provide enough space around the sample for orientation and support, but not be excessively large. Oversized molds increase OCT consumption, prolong OCT solidification time, and may reduce freezing efficiency.

3. Rapid sample handling is critical for preserving RNA quality. All reagents, tools, and sample-handling materials should be prepared in advance and kept cold whenever possible. The sample preparation workflow should be completed as quickly as possible, especially before freezing.

4. Proper sample orientation during OCT embedding is essential. Once the OCT block is frozen, the final sectioning plane cannot be easily corrected. Orientation should therefore be checked carefully under a stereomicroscope before freezing.

5. Section thickness may need to be optimized for different plant tissues. For the samples described here, 12–14 μm sections generally provided intact tissue morphology and reliable section handling. Thinner sections may tear more easily, whereas thicker sections may reduce image clarity or affect downstream transcript capture.

6. Plant tissues often contain air spaces that can interfere with OCT infiltration and section quality. Vacuum infiltration and gradual transfer through ETM can help improve embedding quality, especially for air-rich tissues.

7. For Visium V1, tissue sections are placed directly onto the Visium Gene Expression slide. For Visium HD workflows, sections are placed onto marked tissue slides and later processed using CytAssist. Therefore, the section-position marking and replicate-slide selection steps are especially important for Visium HD workflows.

8. Preparing replicate tissue slides is recommended when possible. Because plant tissue section quality can vary between slides, screening replicate slides before CytAssist transfer helps select the slide with the best morphology, tissue attachment, and section placement.

9. This computational workflow was developed and validated using standard Visium V1 spot-level datasets. Some expression-matrix operations may be adaptable to Visium HD after selecting an appropriate bin size and importing the data, but HD-specific binning, cell segmentation, image registration, quality control thresholds, memory requirements, and spatial plotting have not been validated. Parameters established for Visium V1 should therefore not be transferred directly to Visium HD without independent evaluation.

10. Quality control thresholds should be evaluated separately for each sample and anatomical domain. In the demonstration dataset, genes with fewer than 100 total raw UMI counts were removed, and spots were excluded if either mitochondrial or plastid-derived reads exceeded 5% of their total counts. No additional lower or upper cutoffs for nFeature_RNA or nCount_RNA were applied; their distributions were inspected graphically. These settings reproduce the original analysis and should not be treated as universal recommendations.

11. Monocle 3 pseudotime is defined relative to one or more manually selected SAM-associated principal-graph nodes and does not represent absolute chronological time. Root-node identifiers should be saved and reused for reproducible plotting. The inferred direction should be evaluated against anatomical information and independent biological evidence.

12. SCTransform and Harmony are used for dimensionality reduction, integration, clustering, and visualization. Raw pseudobulk counts, rather than SCT-corrected or Harmony-integrated values, should be used for replicate-level statistical testing.

**Troubleshooting**

**Problem 1:** The OCT block contains cracks or visible ice crystals.

Possible causes: The OCT volume was too large, freezing was too slow, or the isopentane was not sufficiently pre-chilled.

Solutions: Use a smaller custom OCT mold, minimize excess OCT around the sample, and make sure the isopentane is fully pre-chilled before freezing. Immerse the embedding boat immediately after sample orientation.

**Problem 2:** The sample is poorly infiltrated with OCT or contains air gaps.

Possible causes: Air spaces remained inside the plant tissue, or the sample was transferred too quickly through the ETMs.

Solutions: Use vacuum infiltration before OCT embedding and transfer samples gradually through the ETMs. For air-rich tissues, extend the infiltration time slightly, but keep it below 10 min while keeping the sample cold to protect RNA quality.

**Problem 3:** Tissue sections detach during methanol or isopropanol treatment.

Possible causes: Sections were not firmly attached to the slide, liquid was applied too forcefully, or the tissue touched residual liquid in the reagent reservoir.

Solutions: Ensure that sections are firmly attached to the slide before fixation. Handle slides gently during methanol fixation and isopropanol treatment. When removing the isopropanol solution, tilt the slide carefully and avoid contact between the tissue section and any residual liquid. If section detachment still occurs, consider using coated slides, such as poly-L-lysine-coated slides or Schott Nexterion Slide H-3D hydrogel–coated slides, to improve tissue attachment.

**Problem 4:** The computational workflow stops with an out-of-memory error, R closes unexpectedly, or SCTransform and integration take an unusually long time.

Possible causes: Available RAM or temporary disk space is insufficient for the expression matrix and intermediate matrices generated during normalization, PCA, and integration. Compressed RDS size substantially underestimates peak memory use. Automatic restoration of a large .RData workspace or cloud-synchronization activity may further increase resource use.

Solutions: Restart R without restoring .RData, close other memory-intensive applications, and run QC, integration, and downstream analyses as separate processes. Write large temporary and final objects to a local nonsynchronized directory before copying them to cloud storage. For the approximately 20,000-spot Visium V1 demonstration dataset, 16 GB RAM may be marginal, and 32 GB is preferable; these are planning estimates rather than guaranteed requirements. Visium HD requirements must be assessed according to bin size and tissue coverage. If adapting the workflow for limited memory, consider conserve.memory = TRUE, method = "glmGamPoi", sequential rather than parallel processing, larger HD bins, or region-based analysis, and record any resulting parameter changes.

**Problem 5:** Spots do not overlay the correct histological image, or adjacent sections are misregistered in the 3D reconstruction.

Possible causes: The wrong capture-area image or spatial scale was used, section identifiers or section order are incorrect, coordinates were transformed twice, or rotation and reflection were applied inconsistently.

Solutions: Verify each section independently by overlaying spots on its original tissue image and checking recognizable anatomical landmarks. Confirm the image name, coordinate columns, scale factor, section order, and z-position before alignment. Record all rotations, translations, reflections, and cropping operations. Inspect consecutive section pairs before constructing the complete stack. Do not interpret a 3D expression gradient until image and spot registration have been visually confirmed.

**Problem 6:** Samples remain separated after Harmony integration.

Possible causes: Assay layers or biological-replicate labels are incorrect, samples have extreme differences in depth or quality, insufficient biological states are shared among samples, or integration is under- or over-correcting the data.

Solutions: Inspect UMAPs before and after integration, colored separately by biological replicate and structural domain. Confirm that the split RNA/SCT layers correspond to the intended biological replicates and that the same PCA dimensions are used for Harmony, neighbors, and UMAP. Also, consider whether the remaining separation reflects genuine biological differences.
